# Luteolin Exerts Neuroprotection *via* Modulation of the p62/Keap1/Nrf2 Pathway in Intracerebral Hemorrhage

**DOI:** 10.3389/fphar.2019.01551

**Published:** 2020-01-21

**Authors:** Xin Tan, Yi Yang, Jianguo Xu, Peng Zhang, Ruming Deng, Yiguang Mao, Jia He, Yibin Chen, Yan Zhang, Jiasheng Ding, Haiying Li, Haitao Shen, Xiang Li, Wanli Dong, Gang Chen

**Affiliations:** ^1^ Department of Neurology, The First Affiliated Hospital of Soochow University, Suzhou, China; ^2^ Department of Neurosurgery & Brain and Nerve Research Laboratory, The First Affiliated Hospital of Soochow University, Suzhou, China

**Keywords:** intracerebral haemorrhage, luteolin, p62-Keap1-Nrf2 pathway, autophagy, antioxidant, oxidative stress

## Abstract

Upregulation of neuronal oxidative stress is involved in the progression of secondary brain injury (SBI) following intracerebral hemorrhage (ICH). In this study, we investigated the potential effects and underlying mechanisms of luteolin on ICH-induced SBI. Autologous blood and oxyhemoglobin (OxyHb) were used to establish *in vivo* and *in vitro* models of ICH, respectively. Luteolin treatment effectively alleviated brain edema and ameliorated neurobehavioral dysfunction and memory loss *in vivo*. Also, *in vivo*, we found that luteolin promoted the activation of the sequestosome 1 (p62)/kelch‐like enoyl-coenzyme A hydratase (ECH)‐associated protein 1 (Keap1)/nuclear factor erythroid 2-related factor 2 (Nrf2) pathway by enhancing autophagy and increasing the translocation of Nrf2 to the nucleus. Meanwhile, luteolin inhibited the ubiquitination of Nrf2 and increased the expression levels of downstream antioxidant proteins, such as heme oxygenase-1 (HO-1) and reduced nicotinamide adenine dinucleotide phosphate (NADPH): quinine oxidoreductase 1 (NQO1). This effect of luteolin was also confirmed *in vitro*, which was reversed by the autophagy inhibitor, chloroquine (CQ). Additionally, we found that luteolin inhibited the production of neuronal mitochondrial superoxides (MitoSOX) and alleviated neuronal mitochondrial injury *in vitro*, as indicated *via* tetrachloro-tetraethylbenzimidazol carbocyanine-iodide (JC-1) staining and MitoSOX staining. Taken together, our findings demonstrate that luteolin enhances autophagy and anti-oxidative processes in both *in vivo* and *in vitro* models of ICH, and that activation of the p62-Keap1-Nrf2 pathway, is involved in such luteolin-induced neuroprotection. Hence, luteolin may represent a promising candidate for the treatment of ICH-induced SBI.

## Introduction

Intracerebral hemorrhage (ICH) is an important public health problem that has aroused worldwide concern due to its high mortality and morbidity rates (Qureshi et al., 2009). In addition to primary brain injury that disrupts the physical structure of brain tissue, ICH-induced secondary brain injury (SBI) often leads to severe neurological deficits or even death (Xi et al., 2006). Since there has only been minimal progress in the clinical management of ICH, treatment of patients with acute ICH has remained as a challenge for doctors (Law et al., 2017). Therefore, further research is needed for the discovery and development of novel efficacious treatments. There are many pathophysiological changes that have been demonstrated to participate in the process of SBI, including hemoglobin-induced iron overload, oxidative stress, inflammation, cell apoptosis, autophagy, mitochondrial dysfunction, and blood−brain−barrier disruption (Zhou et al., 2014; Duan et al., 2016).

Oxidative stress plays a significant role in ICH-induced SBI. Oxidative stress is involved in pathophysiological processes at multiple stages after ICH (Aronowski and Zhao, 2011). Nuclear factor erythroid-related factor 2 (Nrf2) has been demonstrated to be an important transcription factor that participates in the regulation of oxidative stress and in ameliorating brain damage (Wang et al., 2007; Xu et al., 2017; Zeng and Chen, 2017). Under unstressed states, Nrf2 interacts with its inhibitor, kelch‐like enoyl-coenzyme A hydratase (ECH)‐associated protein 1 (Keap1), to remain in the cytoplasm. Under conditions of oxidative stress, Nrf2 disassociates from Keap1 and translocates to the nucleus to activate the antioxidant response element (ARE), which leads to an increase in the expression of downstream protective proteins such as heme oxygenase-1 (HO-1) and reduced nicotinamide adenine dinucleotide phosphate (NADPH):quinine oxidoreductase-1 (NQO1) (Wang et al., 2018a).

As a lysosomal degradative pathway, autophagy is essential for survival and maintaining cellular homeostasis. In addition, autophagy is involved in diverse diseases and injuries (Jiang et al., 2015), including the pathological processes during ICH (Duan et al., 2017; Li et al., 2018c). Moreover, recent studies have demonstrated that oxidative stress contributes to autophagy (Duan et al., 2016). Additionally, by engulfing or degrading oxidative-stress products, autophagy may have positive effects on reducing oxidative damage (Filomeni et al., 2015), such as *via* reactive oxygen species (ROS)/Nrf2/p62 autophagy (Jiang et al., 2015). As a form of microtubule-associated protein 1A/1B-light chain 3 (LC3), the amount of LC3II is greatly correlated with the formation of autophagosomes and is considered to be an indicator of the extent of autophagy (Kabeya et al., 2000).

As a member of the flavonoid family, luteolin has been shown to exhibit multiple pharmacological effects, such as antioxidative, anti-inflammatory, autophagic-regulatory, apoptotic, and antitumor effects in many disease models (Xiong et al., 2017; Luo et al., 2019; Ma et al., 2019; Yu et al., 2019). Several studies have shown that luteolin exerts neuroprotective effects both *in vitro* and *in vivo* (Xu et al., 2014; Caltagirone et al., 2016; Kwon, 2017; Luo et al., 2019). However, the effects of luteolin on ICH remain poorly understood.

In this study, we investigated the neuroprotective effects of luteolin in ICH-induced SBI, including potential underlying mechanisms related to regulation of antioxidative processes and autophagy. Moreover, we aimed to assess whether luteolin may represent a potential therapeutic candidate for treating ICH.

## Materials and Methods

### Animals

For all pharmacological experiments *in vivo*, adult male Sprague−Dawley rats (250 to 300 g) were purchased from the Animal Center of the Chinese Academy of Sciences (Shanghai, China). The rats had access to water and food *ad libitum* and were group-housed under a 12-h light/dark cycle in animal rooms that had controlled temperature (22 ± 3°C) and humidity (60 ± 5%). All animal experiments were approved by the Ethics Committee of the First Affiliated Hospital of Soochow University. All protocols were in accordance with the National Institutes of Health (NIH) Guide for the Care and Use of Animals.

### Reagents

Anti-HO-1 (ab13243), anti-Nrf2 (ab89443), anti-NQO1 (A18; ab28947), anti-histone H3 (ab1791), anti-ubiquitin (ab7780), anti-SQSTM1/p62 (ab56416), and anti-Keap1 antibodies (ab139729) were purchased from Abcam (Cambridge, MA, USA). Anti-β-actin antibody (sc-376421) and normal mouse immunoglobulin G (IgG) (sc-2025) were purchased from Santa Cruz Biotechnology (Santa Cruz, CA, USA). Anti-β-tubulin (2128L) and anti-LC3B (2775) antibodies were purchased from Cell Signaling Technology (Beverly MA). Protein A + G agarose (P2012), mitochondrial membrane potential assay kits with tetrachloro-tetraethylbenzimidazol carbocyanine iodide (JC-1) (C2006), and ROS assay kits (S0033) were obtained from the Beyotime Institute of Biotechnology (Jiangsu, China). Mitochondrial superoxide (MitoSOX) Red MitoSOX indicator for live-cell imaging (M36008) was purchased from Thermo Fisher Scientific (USA). Luteolin (T1027) was purchased from TargetMol (USA). Horseradish peroxidase (HRP)-conjugated secondary antibodies, anti-rabbit IgG, HRP-linked antibody (7074S), anti-mouse IgG, and HRP-linked antibodies (7076S) were from Cell Signaling Technology (Beverly, MA).

### Induction of Intracerebral Hemorrhage

**Figure 1:**
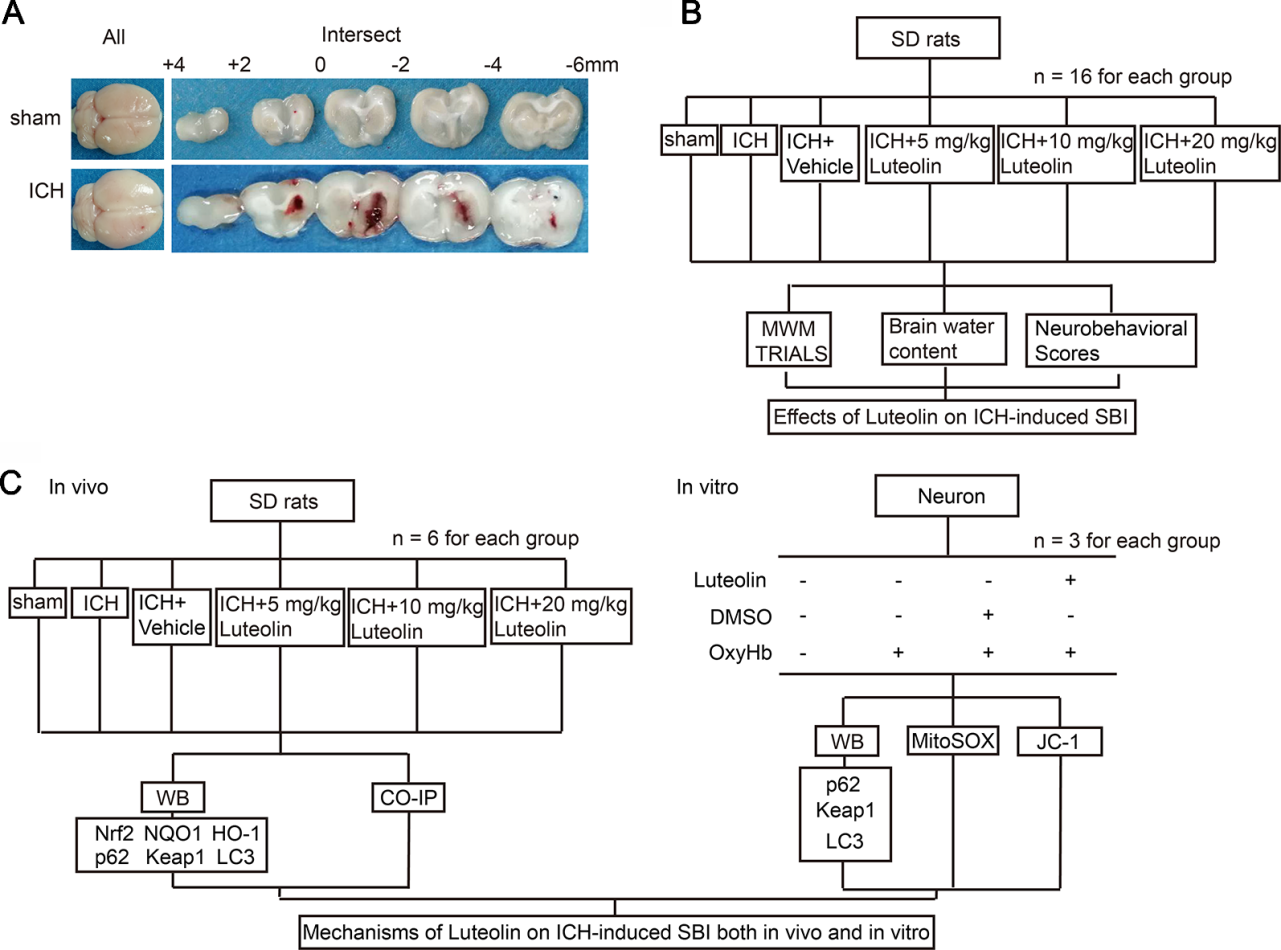
Models of intracerebral hemorrhage (ICH) and experimental designs. **(A)** Coronal brainsections of rats in the sham and ICH groups. **(B)** Effects of luteolin on ICH-inducedsecondary brain injury (SBI) *in vivo*. **(C)** Potential mechanisms of luteolin on ICH-induced SBI both *in vivo* and *in vitro*.

As previously described (Meng et al., 2018), a rat model of ICH was established by injecting 100 μl of autologous blood into the brain of each rat. First, Sprague-Dawley rats were intraperitoneally anesthetized with 4% chloral hydrate and were then mounted onto a stereotactic apparatus (Shanghai Ruanlong Science and Technology Development Co., Ltd., China). After exposing the scalp, we drilled a small hole above the right basal ganglia (1.5 mm posterior to bregma, 3.5 mm lateral to the midline). Then, autologous whole blood, which was collected by cardiac puncturing, was injected slowly (5.5 mm ventral to the cortical surface, at 20 μl/min) with a microliter syringe (Hamilton Company, NV, USA) into the stereotaxically positioned hole above the right basal ganglia. The needle was required to stay in place for 5 min to prevent reflux. Finally, scalp was sutured. Representative brain slices from each group are shown in **Figure 1A**.

### Experimental Design

#### Part I: Potential Effects of Luteolin on Intracerebral Hemorrhage-Induced Secondary Brain Injury in Rats

In this set of experiments, 96 rats (109 rats were used, but only 96 rats ultimately survived) were randomly and equally divided into the following six groups (n = 16 per group): sham group, ICH group, ICH + vehicle group, and three ICH + luteolin treatment groups (i.e., 5, 10, and 20 mg/kg). Rats in the sham group were intracerebrally injected with physiological saline solution (100 μl) into the right basal ganglia at 20 μl/min, after which the microliter syringe stayed in the place for 5 min to prevent reflux. Then, bone wax was used to seal the burr hole and the skin incision was disinfected and sutured, similar to the procedure for rats in the ICH group. ICH-operated rats were injected with 100 μl of autologous whole blood into the right basal ganglia as mentioned above. Luteolin and vehicle [dimethylsulfoxide (DMSO)] were injected intraperitoneally at different intervals (10 min, 24 h, and 48 h after ICH) according to the prescribed dose. First, stock solution of luteolin was prepared. We dissolved 50 mg of luteolin into 1 ml of DMSO to make the stock solution. Next, we diluted the stock solution into the corresponding doses with phosphate buffer saline (PBS). Subsequently, we administered intraperitoneal injections at 10 min, 24 h, and 72 h after surgery. At 24 h after ICH, 10 rats per group were tested for behavioral impairments. At 72 h after ICH, six rats per group were euthanized and their brain tissues were used for detection of brain edema. Finally, another six rats were tested in the Morris water maze on the third, fourth, fifth, and sixth day after surgery to assess changes in cognition (**Figure 1B**).

#### Part II: Potential Mechanisms of Luteolin on Intracerebral Hemorrhage-Induced Secondary Brain Injury *In Vivo*

In this set of experiments, we used a total of 86 rats, among which 72 rats ultimately survived. The surviving 72 rats were randomly divided into six groups with six rats in each group (consistent with the groupings of Part I above). Brain tissues were collected at 24 h after surgery for Western blotting, and coimmunoprecipitation (Co-IP) analysis (**Figure 1C**).

#### Part III: Potential Mechanisms of Luteolin on Oxyhemoglobin-Induced Secondary Brain Injury *In Vitro*

In this set of experiments, primary neurons were cultured and oxyhemoglobin (OxyHb), as a common irritant, was applied to emulate ICH pathophysiology *in vitro*. Primary neurons were treated with different concentrations of luteolin (5, 10, and 20 μM) and OxyHb (10 μM) for 24 h. Finally, Western blotting, JC-1 staining, and MitoSOX staining were performed to assess potential mechanisms of luteolin on OxyHb-induced SBI.

### Neurobehavioral Tests

The effects of luteolin on ICH-induced behavioral impairments were examined by monitoring appetite, locomotor activity, and neurological defects in Sprague-Dawley rats with a scoring system that has been previously published (Li et al., 2018b); **Table 1**). At 24-h post-ICH, 10 rats per group were tested and the data were collected by two investigators blind to the experimental design.

**Table 1:** Neurobehavioral tests.

Category	Behavior	Score
Appetite	Finished meal	0
	Left meal unfinished	1
	Scarcely ate	2
Activity	Walked and reached at least three corners of the cage	0
	Walked with some stimulation	1
	Almost always lying down	2
Deficits	No deficits	0
	Unstable walking	1
	Unable to walk	2

### Brain Water Content

As described in a previous study, at 72 h after ICH brain water content was detected by the dry and wet method (Wang et al., 2018b). In brief, at 72 h after ICH induction, the brain of each rat was harvested immediately. Then the harvested brain tissue was subdivided into the following five parts: cerebellum (CB), ipsilateral cortex (Ipsi-CX), ipsilateral basal ganglia (Ipsi-BG), contralateral basal ganglia (Cont-BG), and contralateral cortex (Cont-CX). The wet weight was recorded immediately after the tissues were weighed with an electronic analytical balance. Subsequently, the dry weight was measured after the samples were dried in a thermostatic drier at 100°C for 72 h. Brain water content was calculated with the following formula: (wet weight − dry weight)/wet weight × 100%.

### Morris Water Maze

As described previously (Shen et al., 2015), the Morris water maze was performed to assess cognitive function in rats. In short, the rats were trained for 3 days (four trials per day) before the ICH surgery was performed. At 3 to 6 days postsurgery (four trials per day), Sprague-Dawley rats were tested in the Morris water maze. The depth of the water tank was half a meter and the diameter was 180 cm. First, the tank was filled with water (20–22°C) to a height of 30 cm, after which ink was added to the water. Black-corded fabric was used to wrap the target platform. The platform, which was 10 cm in diameter, was positioned at 2 cm beneath the surface of the water. The starting location of the rat was altered with each new trial. Moreover, the visual points of reference were kept unchanged around the pool. Each trial was terminated when the rat found the platform or when the trial had lasted for 59 s. Rats were allowed to rest for 20 s on the platform after each trial. During the training phase, the rats were given 1 min to find the platform in the pool. If the rats failed to find the platform, we then guided them to the platform with a rod. The rats were allowed to stay at the platform for 20 s to strengthen their memory before they were removed. The swimming path length, latency, and speed to find the platform for each trial were automatically recorded on a computer. The parameters were used to evaluate learning/memory abilities and cognitive function.

### Western Blotting

After induction of ICH for 24 h, brain samples from the right basal ganglia of each rat were collected and homogenized. Both the brain samples collected and extracted cells (for *in vitro* experiments) were lysed in ice-cold radioimmunoprecipitation assay (RIPA) lysis buffer (Beyotime Institute of Biotechnology, Jiangsu, China). After centrifugation at 12,000 rpm at 4°C for 15 min, the supernatant from each sample was collected. Subsequently, we measured protein concentrations *via* a bicinchoninic (BCA) protein assay kit (Beyotime Institute of Biotechnology). After mixing each sample with sodium dodecyl sulfate (SDS) sample buffer, the protein samples were boiled for 5 min at 100°C. After being separated in a 10% SDS- polyacrylamide gel electrophoresis (PAGE) gel, the protein samples (30 μg per lane) were electrophoretically transferred to a polyvinylidene-difluoride (PVDF) membrane (Millipore Corporation, Billerica, MA, USA), which was then blocked with non-fat milk in PBS-Tween 20 (PBST) for 1 h at room temperature. The membrane was then incubated with primary antibodies overnight at 4°C. The titers of antibodies were as follows: anti-HO-1 antibody (ab13243, 1:1,000 dilution), anti-Nrf2 antibody (ab89443, 1:1,000 dilution), anti-NQO1 antibody (A180; ab28947, 1:1,000 dilution), anti-SQSTM1/p62 antibody (ab56416, 1:1,000 dilution), anti-Keap1 antibody (ab139729, 1:1,000 dilution), LC3B antibody (Cell Signaling Technology, 2775s, 1:1,000 dilution), and anti-ubiquitin antibody (ab7780, 1:1,000 dilution). Furthermore, anti-β-tubulin antibody (Cell Signaling Technology, 2128L, 1:1,000 dilution), anti-histone H3 antibody (ab1791, 1:1,000 dilution), and anti-β-actin antibody (sc-376421, 1:500 dilution) served as loading controls. On the next day, after being washed with PBST (PBS + 0.1% Tween 20), each membrane was incubated with HRP-conjugated secondary antibodies for 1 h at room temperature, after which each membrane was subsequently washed three times with PBST. Protein bands were then revealed *via* an enhanced chemiluminescence (ECL) kit (Beyotime), and protein bands were analyzed *via* ImageJ software (NIH, Bethesda, MD, USA).

### Nuclear and Cytoplasmic Protein Extractions

Nuclear and cytoplasmic proteins were extracted with a nuclear and cytoplasmic protein extraction kit (P0027, Beyotime) according to the manufacturer’s instructions.

### Ubiquitin Analysis

First, the collected brain samples were lysed in ice-cold RIPA lysis buffer. Then the total protein samples were incubated with 1 μg of anti-Nrf2 antibody or IgG (negative control) overnight at 4°C with agitation. Subsequently, the immune complex was incubated with protein A/G agarose beads (Santa Cruz Biotechnology, Santa Cruz, CA) at 4°C for 4 h and was then precipitated under rotary agitation. Finally, the immunoprecipitated proteins were analyzed by SDS−PAGE and immunoblotting with specific antibodies, including anti-Nrf2 and anti-ubiquitin antibodies.

### Cell Culture

As described previously (Sun et al., 2018), primary rat cortical neurons were isolated from 17-day-old rat embryos. In short, we separated the meninges and blood vessels of the brains, which were subsequently rinsed with PBS. Subsequently, the harvested brain tissue was digested with 0.25% trypsin at 37°C for 5 min. The digested brain tissues were then washed with PBS and the resultant brain suspension was centrifuged at 1,500 rpm for 5 min. The resuspended cells were inoculated into 6-well and 12-well plates that were precoated with poly-D-lysine (Sigma, USA). Regarding the inoculation density, we inoculated 2*10^6^ neurons per well into the 6-well plates, and inoculated 1*10^5^ neurons per well into 12-well plates. Subsequently, the dissociated cortical neurons were cultured in Neurobasal medium (Gibco, Carlsbad, CA, USA) that was supplemented with 0.5 mM of GlutaMAX, 2% B-27, 50 U/ml of streptomycin, and 50 U/ml of penicillin (Invitrogen, Grand Island, NY, USA). Finally, the neurons were placed in an incubator at a constant temperature of 37°C and with humidified air containing 5% CO_2_. We changed half of the culture medium every 2 days for 1 week, after which the neurons were harvested for subsequent assays.

### Intracerebral Hemorrhage Models *In Vitro*

We established an *in vitro* of ICH model by using OxyHb which stimulates neurons and induces pathophysiological changes in neurons that are similar to those from ICH. OxyHb (10 μM) was added in Neurobasal medium to stimulate neurons for 24 h at 37°C in 5% CO_2_.

### Tetrachloro-Tetraethylbenzimidazol Carbocyanine Iodide Staining

The mitochondrial membrane potential assay kit was used to detect changes in the mitochondrial membrane potential of neurons, while JC-1 staining was used as an indicator of mitochondrial damage (Beyotime, China), both of which were used according to the manufacturer’s protocol. After being washed with PBS, pretreated neurons were incubated with 1 ml of JC-1 working solution per sample at 37°C for 20 min. Then neurons were washed twice with JC-1 staining buffer. After adding 4′,6-diamidino-2-phenylindole (DAPI) (DAPI Fluoromount-G, SouthernBiotech, USA), we observed the neurons under a fluorescent microscope (Wang et al., 2018b).

### Measurement of Mitochondrial Superoxide

After being treated with OxyHb (10 μM) to mimic ICH *in vitro*, luteolin (10 μM) or vehicle was added into the medium of primary neurons. After 24 h, we firstly prepared the stock solution of 5-mM MitoSOX reagent. Then, 13 μl of DMSO was added to a vial of MitoSOX Red MitoSOX indicator (Thermo Fisher Scientific, USA) containing 50 μg of content. Then, 5-μM MitoSOX reagent working solution was made by diluting the stock solution of 5-mM MitoSOX reagent (mentioned above) with PBS. The neurons of all groups were covered with 5-μM MitoSOX reagent working solution and were incubated at 37°C for 10 min in the dark. Then neurons were mounted in PBS for analysis and imaging after being washed three times with warm PBS.

### Statistical Analysis

We used GraphPad Prism 6 to perform statistical analyses of all experimental data. In addition to neurobehavioral scorings, which are expressed as the median with the interquartile range, all other data are expressed as the mean ± standard deviation (SD). The Mann-Whitney U test was used to analyze neurobehavioral scorings. For all other data, one-or two-way analyses of variance (ANOVAs) were applied to determine significant differences among more than two groups, and we used Tukey’s *post-hoc* tests to determine pairwise differences among the groups. Differences were considered statistically significant at p < 0.05.

## Results

### Luteolin Attenuates Intracerebral Hemorrhage-Induced Secondary Brain Injury *In Vivo*

**Figure 2:**
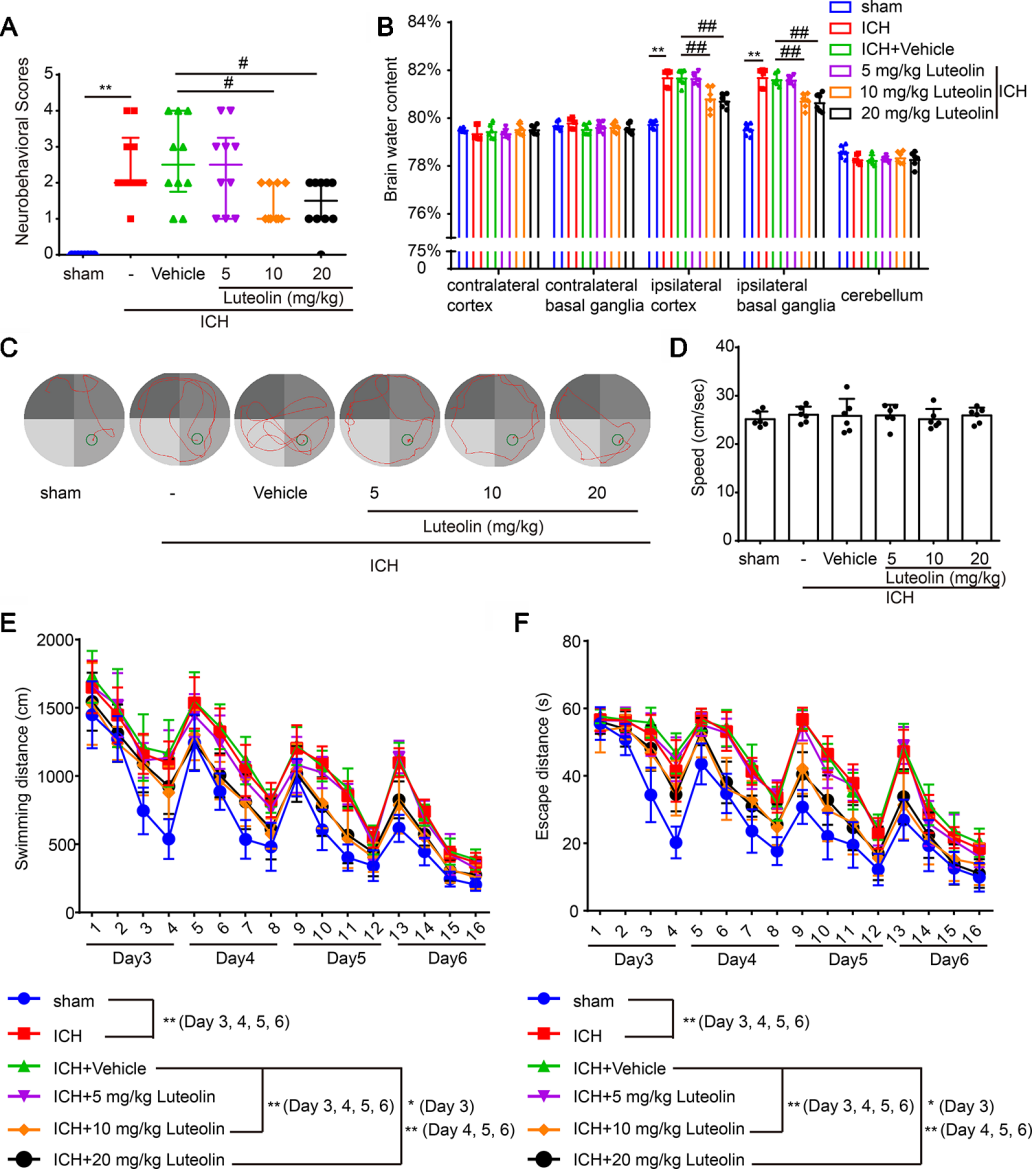
Luteolin ameliorates intracerebral hemorrhage (ICH)-induced neuronal injury. After injection of autologous blood, Sprague-Dawley rats were treated with luteolin (5, 10, 20 mg/kg) or vehicle. **(A)** The neurological scores of rats in the six groups were evaluated and resultant scores are reported in **Table 1** (**p < 0.01 *vs.* sham group; #p < 0.05 *vs.* ICH + vehicle group; n = 10). **(B)** The effects of luteolin on brain water content were examined. All data are shown as the mean ± SD (**p < 0.01 *vs.* sham group; ##p < 0.01 *vs.* ICH + vehicle group; n = 10). **(C**–**F)** Effects of luteolin treatment on cognitive behavioral impairments induced by autologous blood were tested *via* the Morris water maze. **(C)** Representative swimming-path traces of the rats in each group are displayed. **(D)** Swimming speed at the beginning of the test (third-day postsurgery), **(E)** distances and **(F)** escape latencies from four trials per day for a total of 4 days are shown. The values are shown as the mean ± SD (*P < 0.05, **P < 0.01; n = 6).

To evaluate the effect of luteolin on brain injury following ICH, autologous blood was injected into the basal ganglia of rats. Coronal brain sections are shown in **Figure 1A**. Behavioral testing was performed at 24 h after ICH. Damage of neurobehavioral abilities of the ICH group was significantly more severe than that of the sham group, and this impairment was partly alleviated after intraperitoneal injection of 10 mg/kg or 20 mg/kg of luteolin for 24 h (**Figure 2A**). We found that sham group *vs.* ICH group, Z = −4.077, P < 0.0001; ICH + vehicle group *vs.* ICH + 5 mg/kg luteolin group, Z = −0.390, P = 0.8471; ICH + vehicle group *vs.* ICH + 10 mg/kg luteolin group, Z = −2.403, P = 0.0234; ICH + vehicle group *vs.* ICH + 20 mg/kg luteolin group, Z = −2.262, P = 0.024.

Then, we measured brain water content to assess the effect of luteolin on brain edema after ICH. We found that the brain water content was significantly higher in the ICH group compared with that in the sham group in the Ipsi-BG and Ipsi-CX. However, the rise of brain water content in these brain regions was inhibited *via* luteolin (10 or 20 mg/kg). In contrast, there were no significant differences in the brain water content within the Cont-BG, Cont-CX, or CB among the six experimental groups (**Figure 2B**).

In addition, to examine the role of luteolin in cognitive changes induced by ICH, rats were tested in the Morris water maze test (**Figures 2C–F**). Longer escape latencies and swimming distances were observed in rats from the ICH group compared with these parameters in the sham group. In contrast, there were no significant differences in these parameters among the ICH group, and ICH + vehicle group, or ICH + 5 mg/kg luteolin group. However, data from rats in the ICH + 10 mg/kg luteolin group and ICH + 20 mg/kg luteolin group demonstrated that the ICH-induced increases in escape latencies and swimming distances were partially ameliorated *via* luteolin treatments (**Figures 2E, F**). For latencies, the following results were found: third-day postsurgery, F (3, 120) = 88.48, P < 0.0001; on the fourth-day postsurgery, F (3, 120) = 157.4, P < 0.0001; on the fifth-day postsurgery, F (3, 120) = 139.6, P < 0.0001; and on the sixth-day postsurgery, F (3, 120) = 112.4, P < 0.0001. For swimming distance, the following results were found: on the third-day postsurgery, F (3, 120) = 72.53, P < 0.0001; on the fourth-day postsurgery, F (3, 120) = 119.2, P < 0.0001; on the fifth-day postsurgery, F (3, 120) = 115.7, P < 0.0001; and on the sixth-day postsurgery, F (3, 120) = 279.9, P < 0.0001. Additionally, we found there were no significant differences in the swimming speed of all the groups at the beginning of the test (third-day postsurgery) (**Figure 2D**). Overall, luteolin exerted a partial rescuing effect on the brain injury induced by ICH, and this effect was evident at doses of 10 and 20 mg/kg.

### Luteolin Promotes Activation of the Nrf2 Pathway and Enhances Nrf2 Nuclear Translocation Following Intracerebral Hemorrhage *In Vivo*

**Figure 3:**
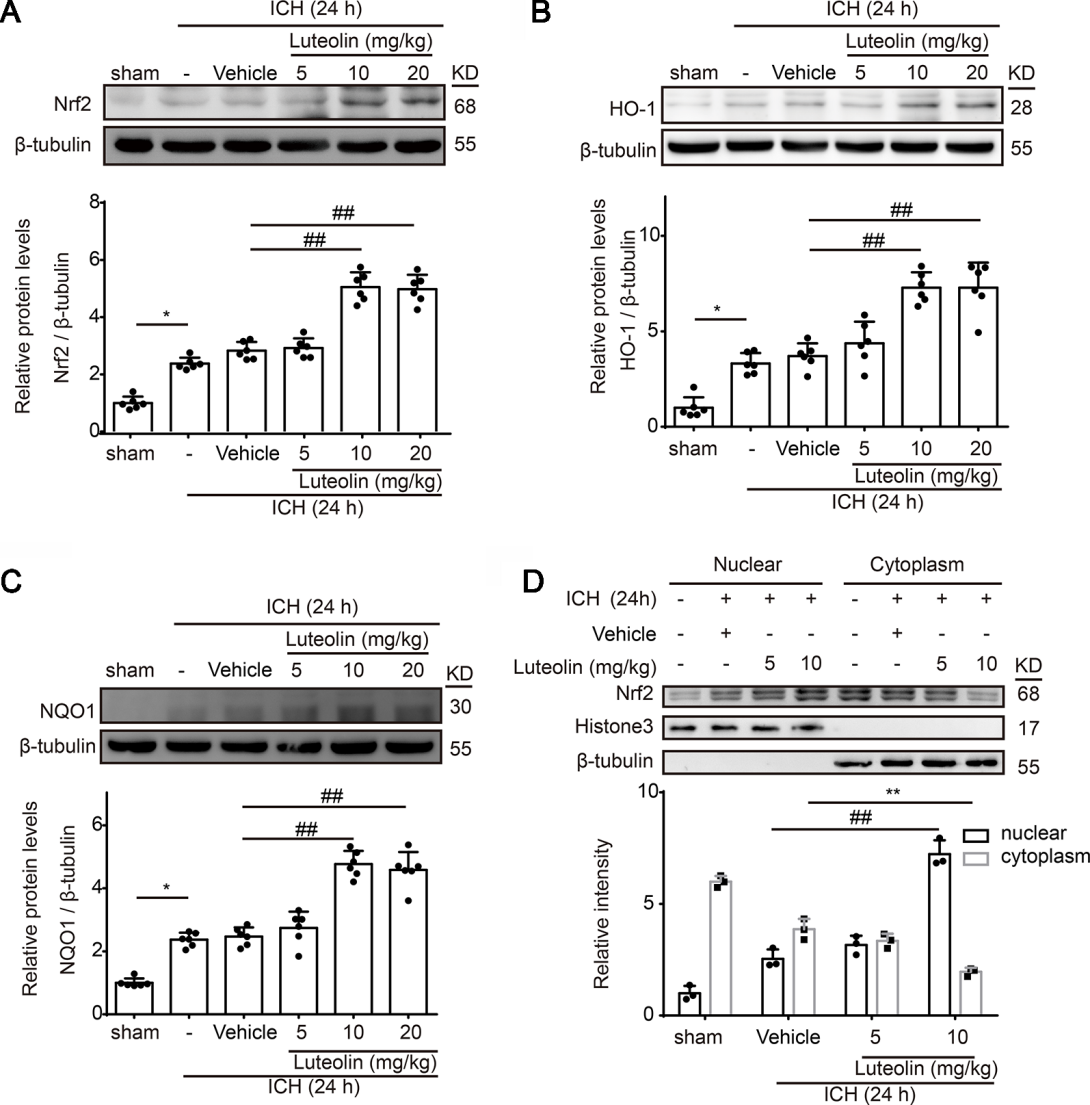
Luteolin treatment promotes intracerebral hemorrhage (ICH)-induced activation of the nuclear factor erythroid 2-related factor 2 (Nrf2) signaling pathway and enhances Nrf2 nuclear translocation. After injecting of autologous blood and luteolin (5, 10, 20 mg/kg), we extracted brain tissue proteins in each group at 1 day after ICH. **(A**–**C)** Protein levels of Nrf2, heme oxygenase-1 (HO-1), and reduced nicotinamide adenine dinucleotide phosphate (NADPH):quinine oxidoreductase 1 (NQO1) were examined by Western blotting. In the quantitative analysis of protein levels, the mean values of proteins in the corresponding sham groups were normalized to 1.0. Data are presented as the mean ± SD (*p < 0.05 *vs.* sham group and ##p < 0.01 *vs.* ICH + vehicle group; n = 6). **(D)** Western-blot analysis of Nrf2 in the nucleus and cytoplasm at 24 h after ICH. Relative protein levels are shown. H3 and β-tubulin served as loading controls. All data are shown as the mean ± SD [##p < 0.01 *vs.* ICH + vehicle group (nuclear); **p < 0.01 *vs.* ICH + vehicle group (cytoplasmic); n = 3]. Full images for Western blots in figures were shown in **Supplementary Material**.

To explore the effects of luteolin on the Nrf2 signaling pathway after ICH, at 24 h after ICH, we detected the protein levels of both Nrf2 and downstream antioxidative proteins of Nrf2 (HO-1 and NQO1) *via* Western blotting. At 24 h after ICH Nrf2 levels were not significantly elevated compared to those of the sham group; however, treatment with luteolin significantly elevated Nrf2 levels at 24 h after ICH (**Figure 3A**). We obtained similar results when we detected the protein levels of HO-1 and NQO1 (**Figures 3B, C**). The promotion effect was only apparent when the dose of luteolin reached 10 and 20 mg/kg. Moreover, to further explore the mechanisms of luteolin on regulating the Nrf2 signaling pathway, we evaluated Nrf2 nuclear translocation by extracting and assaying nuclear and cytoplasmic proteins. As shown in **Figure 3D**, after ICH, nuclear Nrf2 protein levels were increased and corresponded to concomitantly decreased levels of cytoplasmic Nrf2 protein compared to those in the sham group, and this effect was significantly amplified after administration of luteolin (10 mg/kg). These findings suggest that luteolin increased Nrf2 nuclear translocation to activate subsequent pathways at 24 h after ICH, possibly to induce anti-oxidative processes.

### Luteolin Activates the P62-Keap1-Nrf2 Pathway and Enhances Autophagy After Intracerebral Hemorrhage by *In Vivo*

**Figure 4:**
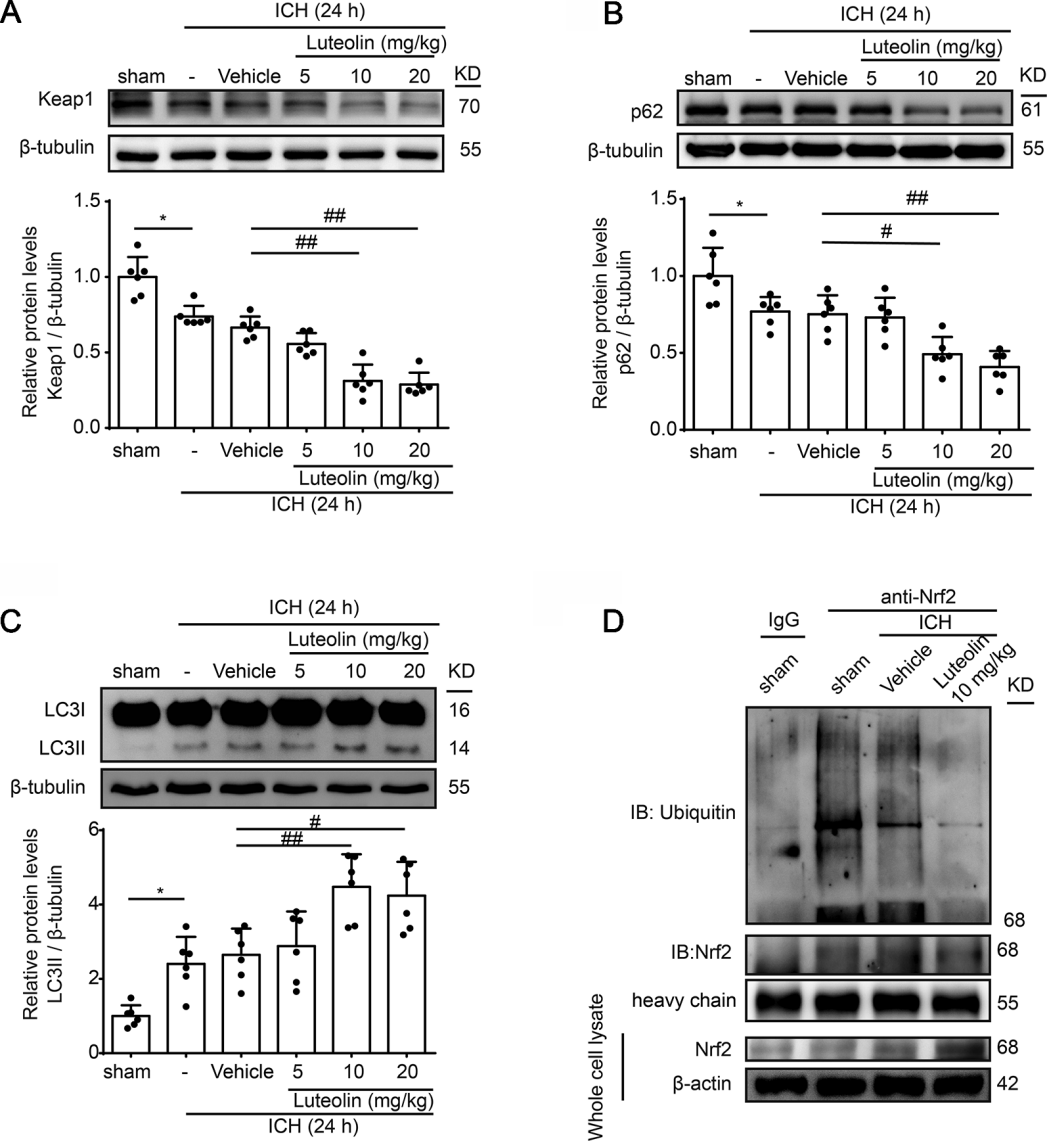
Luteolin promotes autophagy to activate the sequestosome 1 (p62)/kelch‐like enoyl-coenzyme A hydratase (ECH)‐associated protein 1 (Keap1)/nuclear factor erythroid 2-related factor 2 (Nrf2) pathway and inhibits the ubiquitination of Nrf2 *in* *vivo*. Rats were subjected to intracerebral hemorrhage (ICH), and were then injected with luteolin (5, 10, 20 mg/kg) or vehicle. At 24 h after ICH, brain tissues were collected and Western-blot analysis was performed. The protein levels of Keap1 **(A)**, p62 **(B)**, and LC3 **(C)** were evaluated in the six groups. Data are presented as the mean ± SD (*P < 0.05 *vs.* sham group; #P < 0.05, ##P < 0.01 *vs.* ICH + vehicle group; n = 6). **(D)** The interaction between Nrf2 and ubiquitin *in vivo* was analyzed *via* coimmunoprecipitation. Ubiquitin was immunoprecipitated with the anti-Nrf2 antibody and immunoglobulin G (IgG) was used as a negative control. Luteolin inhibited the ubiquitination of Nrf2. Full images for Western blots in figures were shown in **Supplementary Material**.

We next further explored the effects and possible mechanisms of luteolin on the Nrf2 signaling pathway after ICH. Here, we focused on Keap1, which is an important Nrf2 repressor that binds to Nrf2 in the absence of stimulation and is related to ubiquitination of Nrf2 to mediate proteasomal degradation. As shown in **Figure 4A**, the level of Keap1 in brain tissue was decreased at 24 h after ICH compared to that in the sham group but it was not obvious. However, the administration of medium and high doses of luteolin (10, 20 mg/kg) significantly decreased Keap1 levels compared to that in the ICH + vehicle group. p62, which is another type of autophagy-adaptor protein, has been documented to associate with Nrf2 signaling and autophagy *via* binding with Keap1. Finally, p62 sequesters Keap1 into autophagosomes for degradation during autophagy (Jiang et al., 2015). Hence, we next examined the expression of p62 as an indicator of autophagy. As is shown in **Figure 4B**, a lower expression of p62 was found in the ICH group compared with that in the sham group. In contrast, luteolin (10, 20 mg/kg) reduced the expression of p62 compared to that in the ICH + vehicle group. Next, we evaluated the level of LC3II, which is another autophagy-related marker. The expression of LC3II was increased at 24 h after ICH and the treatment of luteolin (10, 20 mg/kg) further increased LC3II expression. This finding suggests that luteolin enhanced autophagy and led to the activation of the downstream Nrf2 signaling pathway (**Figure 4C**).

### Luteolin Protects Intracerebral Hemorrhage-Induced Injury *Via* Inhibition of Nrf2 Ubiquitination *In Vivo*

Reduced Nrf2 ubiquitylation has been recognized to enhance the stability of Nrf2 and to promote the activation of the Nrf2 signaling pathway (Jiang et al., 2015). Therefore, we examined the level of Nrf2 ubiquitination *via* Co-IP assays to further explore the mechanism of luteolin in influencing the Nrf2 signaling pathway. As shown in **Figure 4D**, the interaction between Nrf2 and ubiquitin was obvious in the sham group. Compared with that in the ICH + vehicle group, treatment with luteolin (10 mg/kg) inhibited the interaction between Nrf2 and ubiquitin.

### Luteolin Ameliorates Oxyhemoglobin-Induced Mitochondrial Injury *In Vitro*

**Figure 5:**
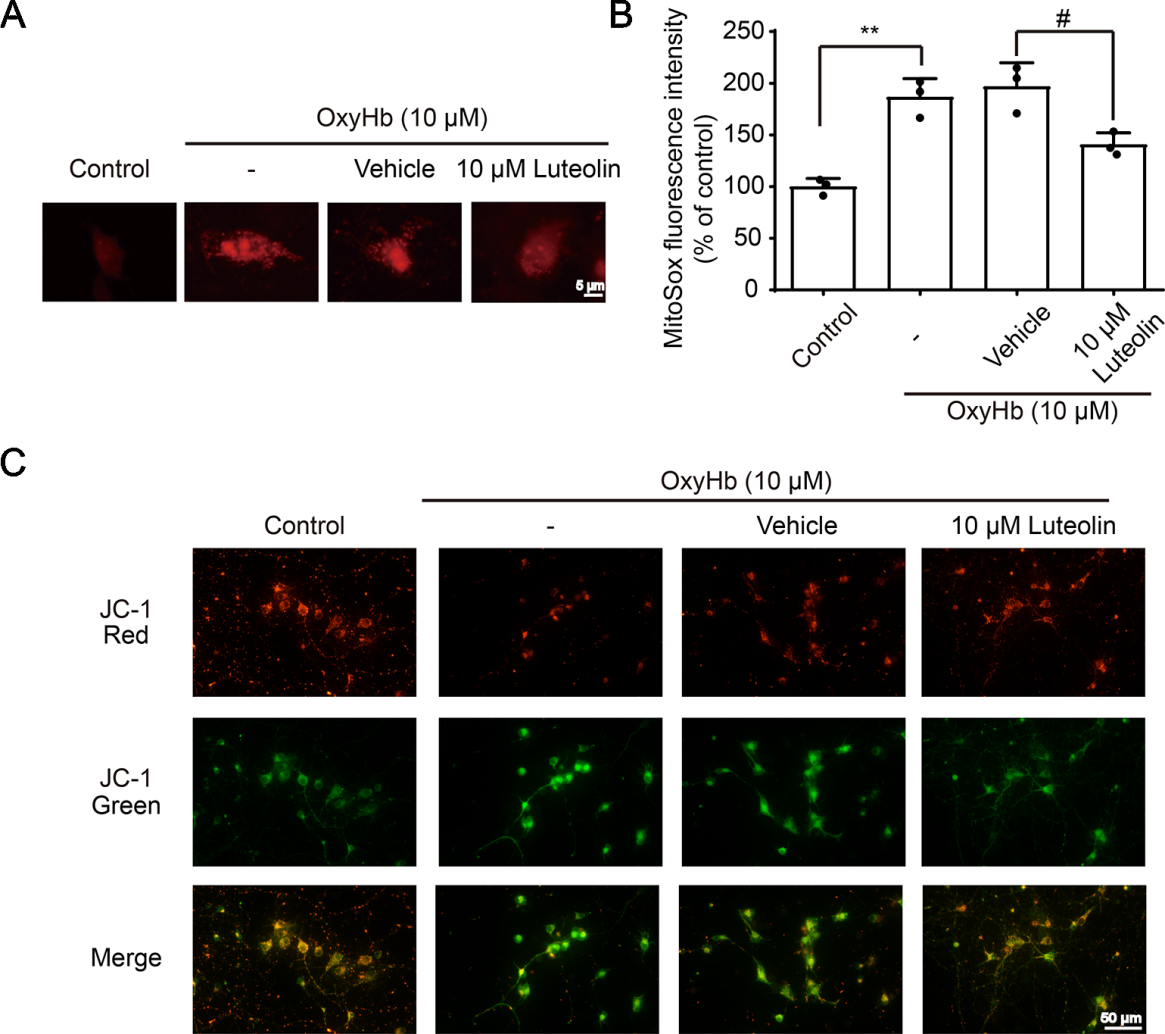
Luteolin attenuates oxyhemoglobin (OxyHb)-induced increases in mitochondrial reactive oxygen species (ROS) and mitochondrial injury *in vitro*. Primary neurons were cultured and were incubated with or without OxyHb (10 μM) and/or co-incubation with luteolin (10 μM) for 24 h. Representative images of mitochondrial superoxide (MitoSOX) staining **(A)**, relative MitoSOX fluorescence intensities analysis **(B)**, and tetrachloro-tetraethylbenzimidazol carbocyanine iodide (JC-1) staining images **(C)** are shown, which indicated the levels of mitochondrial ROS and the degrees of mitochondrial injury. The scale bar = 5 μm **(A)**. Data are presented as the mean ± SD (**P < 0.01 vs. control group; ^#^P < 0.05 vs. OxyHb [10 μM] + vehicle group; n = 3) **(B)**, whereas the scale bar = 50 μm **(C)**.

The Nrf2 signaling pathway has been recognized as a significant pathway for exerting antioxidative processes and downregulating the accumulation of ROS (Zeng and Chen, 2017). As an important indicator for assessing the level of oxidative-stress damage, we used the MitoSOX Red MitoSOX indicator to measure mitochondrial ROS. OxyHb was used to simulate ICH pathophysiology *in vitro* in cultured primary neurons. After being treated with OxyHb (10 μM), using a fluorescent microplate reader, we found increased ROS in the OxyHb group and OxyHb + vehicle group compared to that in the sham group, but this OxyHb-induced increase was inhibited *via* luteolin (10 μM) treatment (**Figures 5A, B**). JC-1 staining is an ideal fluorescent probe for examining changes in the mitochondrial membrane potential (△Ψt). In the absence of stimulation, JC-1 binds to the mitochondrial matrix in the form of J-aggregates, producing red fluorescence. As shown in **Figure 5C**, after treatment with OxyHb, a decrease in red fluorescent intensity and an increase in green fluorescence intensity were observed in the OxyHb group and the OxyHb + vehicle group, which indicated a loss of the mitochondrial membrane potential and openings of mitochondrial permeability transition pores (MPTPs). However, the administration of luteolin (10 μM) reversed such effects. In conclusion, luteolin reduced the production of mitochondrial ROS and played a significant role in mitochondrial protection following OxyHb.

### Luteolin Protects Neurons From Oxyhemoglobin-Induced Injury *via* Activation of the P62/Keap1/Nrf2 Pathway *In Vitro*

**Figure 6:**
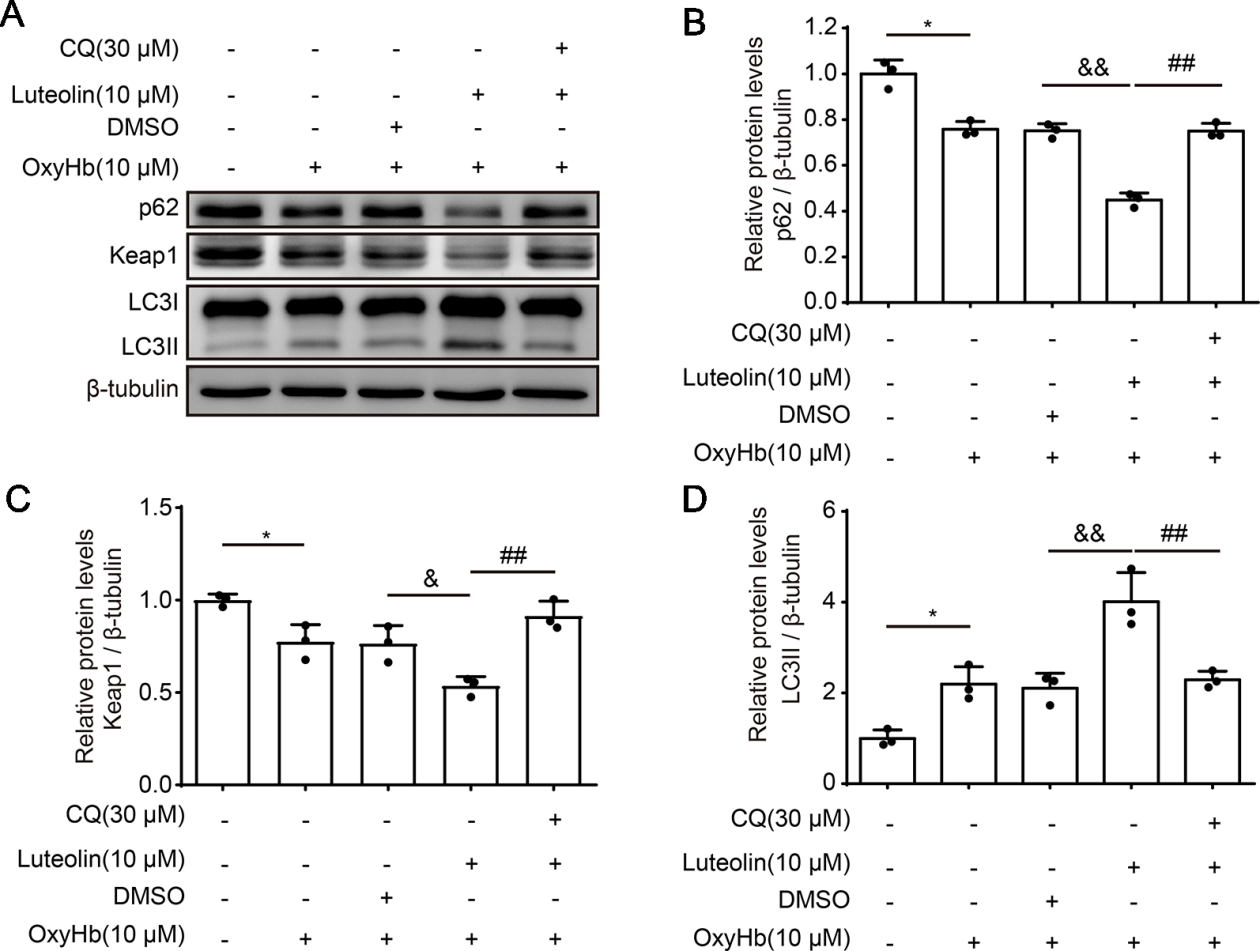
Luteolin promotes the activation of the sequestosome 1 (p62)/kelch‐like enoyl-coenzyme A hydratase (ECH)‐associated protein 1 (Keap1)/nuclear factor erythroid 2-related factor 2 (Nrf2) pathway *in vitro*. **(A)** Western blotting showing that compared to that in the control group, after oxyhemoglobin (OxyHb) stimulation, the protein level of p62 or Keap1 was decreased and the expression of LC3II was increased. Additionally, these changes were magnified with co-treatment of OxyHb (10 μM) and luteolin (10 μM). Moreover, the above changes were reversed *via* chloroquine (CQ). **(B)** Quantification of the protein levels of p62 in the various groups. Data are presented as the mean ± SD [*P < 0.05 *vs.* control group; ^&&^P < 0.01 *vs.* OxyHb (10 μM) + vehicle group; ^##^P < 0.01 *vs.* OxyHb (10 μM) + luteolin (10 μM) group; n = 3]. **(C)** Quantification of the expression of Keap1 in the various groups. Data are presented as the mean ± SD [*P < 0.05 *vs.* control group; ^&^P < 0.05 *vs.* OxyHb (10 μM) + vehicle group; ^##^P < 0.01 *vs.* OxyHb (10 μM) + luteolin (10 μM) group; n = 3]. **(D)** Quantification of the relative levels of LC3II in the various groups. Data are presented as the mean ± SD [*P < 0.05 *vs.* control group; &&P < 0.01 *vs.* OxyHb (10 μM) + vehicle group; ^##^P < 0.01 *vs.* OxyHb (10 μM) + luteolin (10 μM) group; n = 3]. Full images for Western blots in figures were shown in **Supplementary Material**.

To further investigate the role of luteolin in the p62/Keap1/Nrf2 pathway after ICH, we examined the protein levels of p62, Keap1, and LC3II *via* Western blotting of primary neurons *in vitro*. The expression levels of both p62 and Keap1 were decreased after treatment with OxyHb (10 μM), as compared with these levels in the control group. However, the protein levels of both p62 or Keap1 were significantly decreased following co-treatment with OxyHb (10 μM) and luteolin (10 μM), as compared with these levels following OxyHb (10 μM) + vehicle. In order to explore the potential mechanisms of luteolin in the correlation between the p62/Keap1/Nrf2 pathway and autophagy, chloroquine (CQ)—which is an autophagy inhibitor—was used. We found that the luteolin-induced decreases in the protein levels of p62 or Keap1 were reversed after the co-treatment with OxyHb (10 μM), CQ (30 μM), and luteolin (10 μM). Additionally, analysis of LC3II protein levels recapitulated this phenomenon. Pre-treatment with OxyHb (10 μM) and luteolin (10 μM) up-regulated the expression of LC3II, which suggested that there were elevated levels of autophagy, compared with those in the OxyHb (10 μM) + vehicle group. Moreover, this change was reversed *via* CQ (**Figures 6A–D**). In summary, we obtained similar results to those in our *in vivo* experiments, which confirmed the role of luteolin in promoting the activation of the p62/Keap1/Nrf2 pathway.

## Discussion

Over the past several decades, oxidative stress has been found to be involved in the pathogenesis and development of many diseases, including ophthalmic diseases (Nishimura et al., 2017; Pinazo-Duran et al., 2018), diabetes (Rochette et al., 2018), cardiovascular diseases (Luscher, 2015; Munzel et al., 2015; Schiattarella and Hill, 2017), atherosclerosis (Forstermann et al., 2017), arthritis (Bala et al., 2017; Kardes and Karagulle, 2018), dermatological diseases (Rojo de la Vega et al., 2018), respiratory diseases (Hecker, 2018), hepatic diseases (Lee et al., 2019), urinary system diseases (Andersson, 2018), cancer (Poprac et al., 2017), neurodegenerative disorders (Jiang et al., 2016), and other nervous system diseases (D’Amico et al., 2013; Patel, 2016; Pei and Fan, 2017). Moreover, oxidative stress also participates in pathological processes after ICH (Aronowski and Zhao, 2011). Inhibition of oxidative stress has been demonstrated to improve the prognosis of ICH, ameliorate neurobehavioral impairments, and reduce brain edema (Wei et al., 2017; Zeng and Chen, 2017; Sosa et al., 2018; Wang et al., 2018b). Autophagy is involved in the pathophysiological processes of various diseases, as well as in ICH (He et al., 2008). In recent years, the crosstalk between autophagy and anti-oxidative processes has received considerable attention, and related studies have suggested that autophagy may enhance antioxidative processes in a variety of disease models (Giordano et al., 2014; He et al., 2017; Li et al., 2018a).

ICH exhibits high disability and mortality rates. Moreover, ICH has become a heavy burden for global health care systems and societies (Selim et al., 2019). Supportive medical care has represented the main treatment for ICH but has yielded an insufficient efficacy (Hanley et al., 2019). Numerous studies have been carried out in order to further investigate the mechanisms of ICH-induced SBI. Many kinds of recombinant proteins, compounds, drugs, and other agents—including recombinant complement component 1q (C1q)/tumor necrosis factor (TNF)-related protein 9 (rCTRR9), recombinant osteopontin (rOPN), isoliquiritigenin, andrographolide, and melatonin—have been reported to exert neuroprotective effects after ICH by alleviating brain injury, inhibiting neuronal apoptosis, suppressing oxidative stress, down-regulating inflammatory damage, and protecting the blood−brain barrier (Zeng and Chen, 2017; Gong et al., 2018; Li et al., 2018d; Wang et al., 2018b; Zhao et al., 2018). However, the protective effects of these neuroprotective agents are still lacking in clinical applications, and many such agents include problematic side effects. Therefore, there is a continued need to further identify and develop novel drugs that are both safe and efficacious in the treatment of ICH.

Luteolin is a member of the naturally occurring flavonoid family and has various beneficial bioactivities. Numerous studies have revealed anti-inflammatory, antioxidative, anti-apoptotic, autophagic-regulatory, anti-viral, anticancer, and metabolic effects of luteolin, which have been confirmed in many different disease models (Hu et al., 2016; Zhang et al., 2016; Peng et al., 2017; Du et al., 2018; Liu et al., 2018; Tan et al., 2018b; Yang et al., 2018b; Kang et al., 2019). In addition, in studies on ischemic stroke (Qiao et al., 2012; Tan et al., 2018a; Luo et al., 2019), traumatic brain injury (Xu et al., 2014), neurodegenerative diseases (Kwon, 2017; Zhang et al., 2017), and other neurological diseases, luteolin has been shown to exert therapeutic effects. Compared with other properties of agents, luteolin has a wide range of sources and is cost-effective. Moreover, because of its lipophilicity, luteolin is able to freely penetrate the blood-brain barrier even if it is administered peripherally (Sawmiller et al., 2014). However, to the best of our knowledge, the impact of luteolin on ICH-induced SBI has remained unclear. Hence, our study focused on this direction and attempted to elucidate any underlying mechanisms.

In this study, we demonstrated that luteolin enhanced the activation of the Nrf2 pathway and enhanced Nrf2 nuclear translocation after ICH. Nrf2 is known to regulate various antioxidant enzymes to protect cells against oxidative stress and is essential for the clearance of hematomas (Zhao et al., 2015; Kang et al., 2019). Numerous studies have demonstrated that activation of the Nrf2 signaling pathway is beneficial in alleviating ICH-induced SBI. Additionally, activation of the Nrf2 signaling pathway has been suggested to be an underlying mechanism related to the efficacies of other agents in ICH treatment (Lan et al., 2017; Wei et al., 2017; Zeng and Chen, 2017). Moreover, previous studies have revealed that luteolin upregulates Nrf2 expression and triggers Nrf2 translocation in various disease models, including brain diseases (Xu et al., 2014; Liu et al., 2018; Tan et al., 2018b; Ma et al., 2019). Our present findings were consistent with those of the previous studies.

Previous studies have revealed that there are differential effects of luteolin on the Nrf2 signaling pathway in different cell lines. For example, luteolin was recognized as an Nrf2 inhibitor and suppressed the activity of the Nrf2/ARE pathway in human lung carcinoma A549 cells (Tang et al., 2011). Son et al. found that luteolin has a bidirectional regulation of the Nrf2 pathway at different stages of disease development (Son et al., 2017). These findings indicate that the role of luteolin in the regulation of the Nrf2/ARE pathway may be different in different cell types. At the same, the biological timing context may be a significant factor.

**Figure 7:**
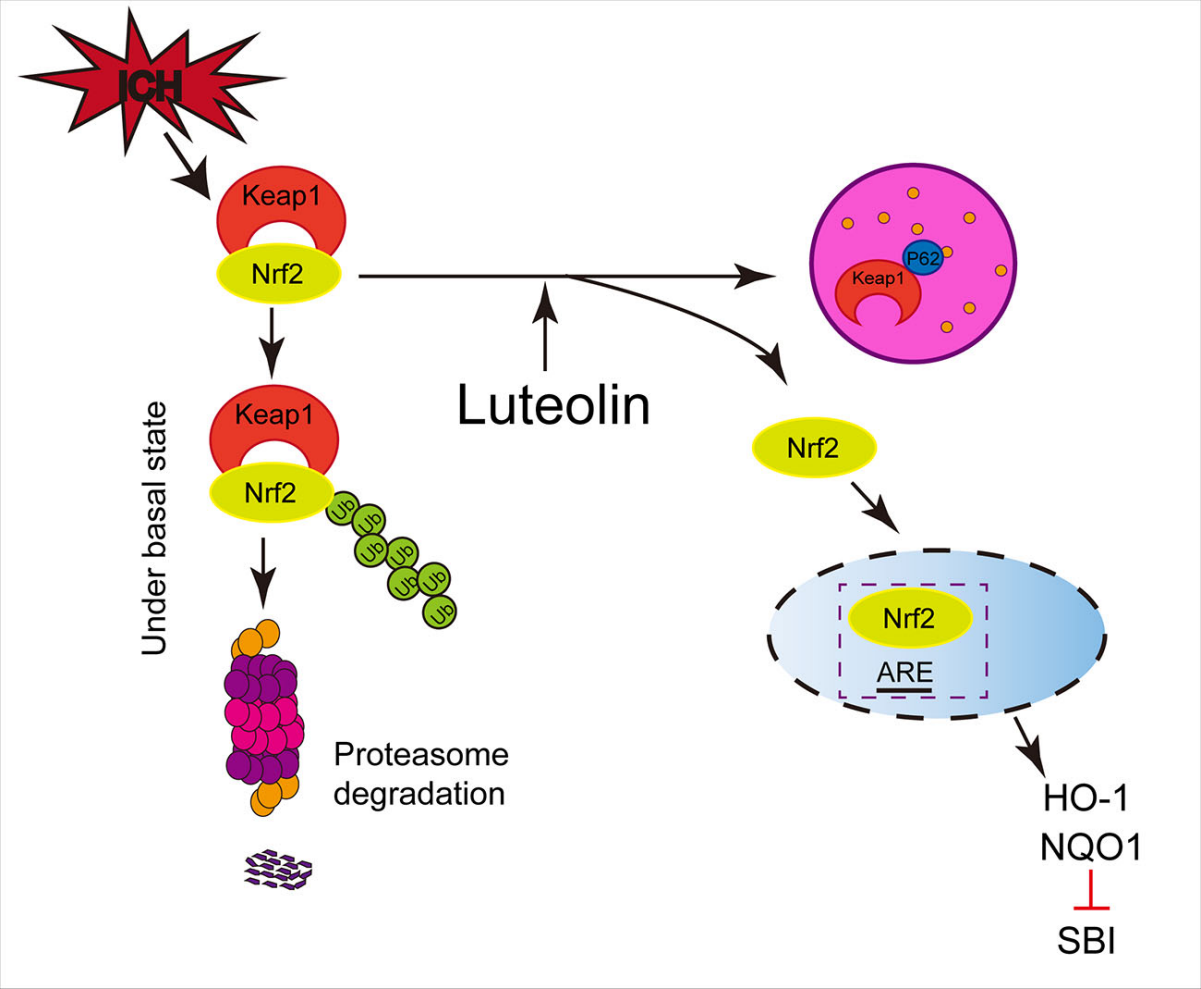
Potential mechanisms of luteolin in ameliorating intracerebral hemorrhage (ICH)-induced secondary brain injury (SBI). Luteolin enhances autophagy, activates the sequestosome 1 (p62)/kelch‐like enoyl-coenzyme A hydratase(ECH)‐associated protein 1 (Keap1)/nuclear factor erythroid 2-related factor 2 (Nrf2) pathway, and plays an important role in neuroprotection and anti-oxidative processes, which suggests that luteolin may represent a promising drug for ameliorating ICH-induced SBI.

As a type of LC3-binding protein, p62 functions as a critical autophagy-adaptor protein and promotes the selective degradation of proteins *via* autophagy (Komatsu et al., 2007). The results of related studies have indicated that by physically isolating Keap1 and impairing the ubiquitylation of Nrf2, p62 mediates the activation of Nrf2 and its downstream pathways and plays an important role in antioxidative processes (Jiang et al., 2015). In the process of exploring new drugs, up-regulation of autophagy has been found in numerous disease-model studies (Yang et al., 2018a; Rusmini and Cortese, 2019) and activation of the p62/Keap1/Nrf2 pathway has been shown to play a role in alleviating systemic diseases (Sun et al., 2016; Su et al., 2018) and in ameliorating brain injury, such as ischemic stroke (Wu et al., 2019). Our present findings were similar to those described above. In addition, luteolin has been found to enhance autophagy in studies of other diseases (Xu et al., 2014; Hu et al., 2016; Cao et al., 2017). Additionally, the administration of autophagic inhibitors such as CQ, has been shown to be associated with exacerbating disease progression (Yang et al., 2018a; Wu et al., 2019). These findings are consistent with our present results, such that we found that luteolin enhanced autophagy and activated the p62/Keap1/Nrf2 pathway, and this effect was reversed by the autophagic inhibitor, CQ, in our ICH model (**Figure 7**).

Above all, our findings suggest that luteolin may represent a novel treatment for ICH-induced SBI. However, our study had some limitations. Our study used Sprague−Dawley male rats as animal models. However, in clinical epidemiological studies, there are also female patients with ICH, and the incidence of ICH in elderly patients is higher than in younger patients. Furthermore, the specific details of luteolin promoting autophagy and affecting the p62/Keap1/Nrf2 pathway remain unclear.

## Conclusion

Taken together, our results demonstrate that autophagy increases slightly after ICH, which activates the p62/Keap1/Nrf2 pathway and upregulates the expression levels of its downstream antioxidant proteins, HO-1 and NQO1, but that effect was not obvious. In contrast, the administration of luteolin significantly amplified the above effects and may have the potential to attenuate ICH-induced SBI in ICH patients.

## Data Availability Statement

The raw data supporting the conclusions of this article will be made available by the authors, without undue reservation, to any qualified researcher.

## Ethics Statement

All animal experiments were approved by the Ethics Committee of the First Affiliated Hospital of Soochow University. All protocols were in accordance with the National Institutes of Health Guide for the Care and Use of Animals.

## Author Contributions

WD and XL were responsible for the conception and design of the experiments. XT, YY, and JX performed the experiments. PZ, RD, YM, JH, YC, YZ and JD participated in data analysis. XT wrote the manuscript. HL, HS, GC, and YY was responsible for its revision. All the authors read and approved the final version of the manuscript for publication.

## Funding

This work was supported by the Project of Jiangsu Provincial Medical Innovation Team (No. CXTDA2017003), Suzhou Key Medical Centre (No. Szzx201501), Scientific Department of Jiangsu Province (No. BE2017656), the Natural Science Foundation of Jiangsu Province (Grants No. BK20170371 and BK20180204), Suzhou Government (No. LCZX201601), and the National Key R&D Program of China (No. 2018YFC1312600 and No. 2018YFC1312601).

## Conflict of Interest

The authors declare that the research was conducted in the absence of any commercial or financial relationships that could be construed as a potential conflict of interest.

## Abbreviations

ICH, intracerebral hemorrhage; SBI, secondary brain injury; SD, Sprague-Dawley; OxyHb, oxyhemoglobin; Co-IP, co-immunoprecipitation; PBS, phosphate buffer saline; LC3, microtubule-associated protein 1A/1B-light chain 3; p62, sequestosome 1; Keap1, kelch‐like ECH‐associated protein 1; Nrf2, nuclear factor erythroid 2-related factor 2; HO-1, heme oxygenase-1; NQO1, NADPH, quinine oxidoreductase 1; CQ, chloroquine; ARE, antioxidant response element; ROS, reactive oxygen species; JC-1, tetrachloro-tetraethylbenzimidazol carbocyanine iodide; MitoSOX, mitochondrial superoxide; HRP, horseradish peroxidase; CB, the cerebellum; Ipsi-CX, ipsilateral cortex; Ipsi-BG, the ipsilateral basal ganglia; Cont-BG, the contralateral basal ganglia; Cont-CX, the contralateral basal cortex; PVDF, polyvinylidene difluoride; DCF-DA, 2,7-dichlorofluorescein diacetate.

## Supplementary Material

The Supplementary Material for this article can be found onlineat : https://www.frontiersin.org/articles/10.3389/fphar.2019.01551/full#supplementary-material

## References

[B1] Andersson K. E. (2018). Oxidative stress and its possible relation to lower urinary tract functional pathology. B.J.U. Int. 121 (4), 527–533. 10.1111/bju.14063 29063681

[B2] Aronowski J. Zhao X. (2011). Molecular pathophysiology of cerebral hemorrhage: secondary brain injury. Stroke 42 (6), 1781–1786. 10.1161/strokeaha.110.596718 21527759 PMC3123894

[B3] Bala A. Mondal C. Haldar P. K. Khandelwal B. (2017). Oxidative stress in inflammatory cells of patient with rheumatoid arthritis: clinical efficacy of dietary antioxidants. Inflammopharmacology 25 (6), 595–607. 10.1007/s10787-017-0397-1 28929423

[B4] Caltagirone C. Cisari C. Schievano C. Di Paola R. Cordaro M. Bruschetta G. (2016). Co-ultramicronized Palmitoylethanolamide/Luteolin in the Treatment of Cerebral Ischemia: from Rodent to Man. Transl. Stroke Res. 7 (1), 54–69. 10.1007/s12975-015-0440-8 26706245 PMC4720704

[B5] Cao Z. Zhang H. Cai X. Fang W. Chai D. Wen Y. (2017). Luteolin promotes cell apoptosis by inducing autophagy in Hepatocellular Carcinoma. Cell Physiol. Biochem. 43 (5), 1803–1812. 10.1159/000484066 29049999

[B6] D’Amico E. Factor-Litvak P. Santella R. M. Mitsumoto H. (2013). Clinical perspective on oxidative stress in sporadic amyotrophic lateral sclerosis. Free Radic. Biol. Med. 65, 509–527. 10.1016/j.freeradbiomed.2013.06.029 23797033 PMC3859834

[B7] Du Y. Liu P. Xu T. Pan D. Zhu H. Zhai N. (2018). Luteolin Modulates SERCA2a leading to attenuation of Myocardial Ischemia/Reperfusion injury *via* Sumoylation at Lysine 585 in Mice. Cell Physiol. Biochem. 45 (3), 883–898. 10.1159/000487283 29421780

[B8] Duan X. Wen Z. Shen H. Shen M. Chen G. (2016). Intracerebral hemorrhage, oxidative stress, and antioxidant therapy. Oxid. Med. Cell Longev. 2016, 1203285. 10.1155/2016/1203285 27190572 PMC4848452

[B9] Duan X. C. Wang W. Feng D. X. Yin J. Zuo G. Chen D. D. (2017). Roles of autophagy and endoplasmic reticulum stress in intracerebral hemorrhage-induced secondary brain injury in rats. CNS Neuroscience Therapeutics 23, 7, 554–566. 10.1111/cns.12703 28544790 PMC6492729

[B10] Filomeni G. De Zio D. Cecconi F. (2015). Oxidative stress and autophagy: the clash between damage and metabolic needs. Cell Death Differ. 22 (3), 377–388. 10.1038/cdd.2014.150 25257172 PMC4326572

[B11] Forstermann U. Xia N. Li H. (2017). Roles of Vascular Oxidative Stress and Nitric Oxide in the Pathogenesis of Atherosclerosis. Circ. Res. 120 (4), 713–735. 10.1161/circresaha.116.309326 28209797

[B12] Giordano S. Darley-Usmar V. Zhang J. (2014). Autophagy as an essential cellular antioxidant pathway in neurodegenerative disease. Redox Biol. 2, 82–90. 10.1016/j.redox.2013.12.013 24494187 PMC3909266

[B13] Gong L. Manaenko A. Fan R. Huang L. Enkhjargal B. McBride D. (2018). Osteopontin attenuates inflammation *via* JAK2/STAT1 pathway in hyperglycemic rats after intracerebral hemorrhage. Neuropharmacology 138, 160–169. 10.1016/j.neuropharm.2018.06.009 29885817 PMC6487497

[B14] Hanley D. F. Thompson R. E. Rosenblum M. Yenokyan G. Lane K. McBee N. (2019). Efficacy and safety of minimally invasive surgery with thrombolysis in intracerebral haemorrhage evacuation (MISTIE III): a randomised, controlled, open-label, blinded endpoint phase 3 trial. Lancet 393 (10175), 1021–1032. 10.1016/s0140-6736(19)30195-3 30739747 PMC6894906

[B16] He Y. Wan S. Hua Y. Keep R. F. Xi G. (2008). Autophagy after experimental intracerebral hemorrhage. J. Cereb. Blood Flow Metab. 28 (5), 897–905. 10.1038/sj.jcbfm.9600578 17987045

[B15] He Y. Li S. Zhang W. Dai W. Cui T. Wang G. (2017). Dysregulated autophagy increased melanocyte sensitivity to H2O2-induced oxidative stress in vitiligo. Sci. Rep. 7, 42394. 10.1038/srep42394 28186139 PMC5301258

[B17] Hecker L. (2018). Mechanisms and consequences of oxidative stress in lung disease: therapeutic implications for an aging populace. Am. J. Physiol. Lung Cell Mol. Physiol. 314 (4), L642–l653. 10.1152/ajplung.00275.2017 29351446 PMC5966777

[B18] Hu J. Man W. Shen M. Zhang M. Lin J. Wang T. (2016). Luteolin alleviates post-infarction cardiac dysfunction by up-regulating autophagy through Mst1 inhibition. J. Cell Mol. Med. 20 (1), 147–156. 10.1111/jcmm.12714 26538370 PMC4717847

[B19] Jiang T. Harder B. Rojo de la Vega M. Wong P. K. Chapman E. Zhang D. D. (2015). p62 links autophagy and Nrf2 signaling. Free Radic. Biol. Med. 88 (Pt B), 199–204. 10.1016/j.freeradbiomed.2015.06.014 26117325 PMC4628872

[B20] Jiang T. Sun Q. Chen S. (2016). Oxidative stress: a major pathogenesis and potential therapeutic target of antioxidative agents in Parkinson’s disease and Alzheimer’s disease. Prog. Neurobiol. 147, 1–19. 10.1016/j.pneurobio.2016.07.005 27769868

[B21] Kabeya Y. Mizushima N. Ueno T. Yamamoto A. Kirisako T. Noda T. (2000). LC3, a mammalian homologue of yeast Apg8p, is localized in autophagosome membranes after processing. EMBO J. 19 (21), 5720–5728. 10.1093/emboj/19.21.5720 11060023 PMC305793

[B22] Kang K. A. Piao M. J. Hyun Y. J. Zhen A. X. Cho S. J. Ahn M. J. (2019). Luteolin promotes apoptotic cell death *via* upregulation of Nrf2 expression by DNA demethylase and the interaction of Nrf2 with p53 in human colon cancer cells. Exp. Mol. Med. 51 (4), 40. 10.1038/s12276-019-0238-y 30988303 PMC6465248

[B23] Kardes S. Karagulle M. (2018). Association of oxidative stress with clinical characteristics in patients with rheumatoid arthritis. Eur. J. Clin. Invest. 48(1), e12858. 10.1111/eci.12858 29144558

[B24] Komatsu M. Waguri S. Koike M. Sou Y. S. Ueno T. Hara T. (2007). Homeostatic levels of p62 control cytoplasmic inclusion body formation in autophagy-deficient mice. Cell 131 (6), 1149–1163. 10.1016/j.cell.2007.10.035 18083104

[B25] Kwon Y. (2017). Luteolin as a potential preventive and therapeutic candidate for Alzheimer’s disease. Exp. Gerontol. 95, 39–43. 10.1016/j.exger.2017.05.014 28528007

[B26] Lan X. Han X. Li Q. Wang J. (2017). (-)-Epicatechin, a natural flavonoid compound, protects astrocytes against hemoglobin toxicity *via* Nrf2 and AP-1 signaling pathways. Mol. Neurobiol. 54 (10), 7898–7907. 10.1007/s12035-016-0271-y 27864733 PMC5436959

[B27] Law Z. K. Appleton J. P. Bath P. M. Sprigg N. (2017). Management of acute intracerebral haemorrhage - an update. Clin. Med. (London England) 17 (2), 166–172. 10.7861/clinmedicine.17-2-166 PMC6297624 28365631

[B28] Lee E. Lim Y. Kwon S. W. Kwon O. (2019). Pinitol consumption improves liver health status by reducing oxidative stress and fatty acid accumulation in subjects with non-alcoholic fatty liver disease: a randomized, double-blind, placebo-controlled trial. J. Nutr. Biochem. 68, 33–41. 10.1016/j.jnutbio.2019.03.006 31030165

[B29] Li C. Jiang F. Li Y. L. Jiang Y. H. Yang W. Q. Sheng J. (2018a). Rhynchophylla total alkaloid rescues autophagy, decreases oxidative stress and improves endothelial vasodilation in spontaneous hypertensive rats. Acta Pharmacol. Sin. 39 (3), 345–356. 10.1038/aps.2017.120 29119967 PMC5843829

[B30] Li H. Liu S. Sun X. Yang J. Yang Z. Shen H. (2018b). Critical role for Annexin A7 in secondary brain injury mediated by its phosphorylation after experimental intracerebral hemorrhage in rats. Neurobiol. Dis. 110, 82–92. 10.1016/j.nbd.2017.11.012 29196215

[B31] Li H. Wu J. Shen H. Yao X. Liu C. Pianta S. (2018c). Autophagy in hemorrhagic stroke: Mechanisms and clinical implications. Prog. Neurobiol. 163–164, 79–97. 10.1016/j.pneurobio.2017.04.002 28414101

[B32] Li X. Wang T. Zhang D. Li H. Shen H. Ding X. (2018d). Andrographolide ameliorates intracerebral hemorrhage induced secondary brain injury by inhibiting neuroinflammation induction. Neuropharmacology 141, 305–315. 10.1016/j.neuropharm.2018.09.015 30218674

[B33] Liu B. Yu H. Baiyun R. Lu J. Li S. Bing Q. (2018). Protective effects of dietary luteolin against mercuric chloride-induced lung injury in mice: involvement of AKT/Nrf2 and NF-kappaB pathways. Food Chem. Toxicol. 113, 296–302. 10.1016/j.fct.2018.02.003 29421646

[B34] Luo S. Li H. Mo Z. Lei J. Zhu L. Huang Y. (2019). Connectivity map identifies luteolin as a treatment option of ischemic stroke by inhibiting MMP9 and activation of the PI3K/Akt signaling pathway. Exp. Mol. Med. 51 (3), 37. 10.1038/s12276-019-0229-z 30911000 PMC6434019

[B35] Luscher T. F. (2015). Ageing, inflammation, and oxidative stress: final common pathways of cardiovascular disease. Eur. Heart J. 36 (48), 3381–3383. 10.1093/eurheartj/ehv679 26690751

[B37] Ma B. Zhang J. Zhu Z. Zhao A. Zhou Y. Ying H. (2019). Luteolin ameliorates testis injury and blood-testis barrier disruption through the Nrf2 signaling pathway and by upregulating Cx43. Mol. Nutr. Food Res. 63 (10), e1800843. 10.1002/mnfr.201800843 30924608

[B38] Meng C. Zhang J. Dang B. Li H. Shen H. Li X. (2018). PERK Pathway activation promotes intracerebral hemorrhage induced secondary brain injury by inducing neuronal apoptosis both in vivo and in vitro. Front. Neurosci. 12, 111. 10.3389/fnins.2018.00111 29541018 PMC5835756

[B39] Munzel T. Gori T. Keaney J. F. Jr. Maack C. Daiber A. (2015). Pathophysiological role of oxidative stress in systolic and diastolic heart failure and its therapeutic implications. Eur. Heart J. 36 (38), 2555–2564. 10.1093/eurheartj/ehv305 26142467 PMC7959410

[B40] Nishimura Y. Hara H. Kondo M. (2017). Oxidative stress in retinal diseases. Oxid Med Cell Longev. 2017, 4076518. 10.1155/2017/4076518 28424744 PMC5382422

[B41] Patel M. (2016). Targeting oxidative stress in central nervous system disorders. Trends Pharmacol. Sci. 37 (9), 768–778. 10.1016/j.tips.2016.06.007 27491897 PMC5333771

[B42] Pei J. P. Fan L. H. (2017). HSYA alleviates secondary neuronal death through attenuating oxidative stress, inflammatory response, and neural apoptosis in SD rat spinal cord compression injury. J. Neuroinflammation. 14, 1, 97. 10.1186/s12974-017-0870-1 28468657 PMC5415746

[B43] Peng M. Watanabe S. Chan K. W. K. He Q. Zhao Y. Zhang Z. (2017). Luteolin restricts dengue virus replication through inhibition of the proprotein convertase furin. Antiviral Res. 143, 176–185. 10.1016/j.antiviral.2017.03.026 28389141

[B44] Pinazo-Duran M. D. Shoaie-Nia K. Zanon-Moreno V. Sanz-Gonzalez S. M. Del Castillo J. B. Garcia-Medina J. J. (2018). Strategies to reduce oxidative stress in glaucoma patients. Curr. Neuropharmacol. 16 (7), 903–918. 10.2174/1570159x15666170705101910 28677495 PMC6120109

[B45] Poprac P. Jomova K. Simunkova M. Kollar V. Rhodes C. J. Valko M. (2017). Targeting Free Radicals in Oxidative Stress-Related Human Diseases. Trends Pharmacol. Sci. 38 (7), 592–607. 10.1016/j.tips.2017.04.005 28551354

[B46] Qiao H. Zhang X. Zhu C. Dong L. Wang L. Zhang X. (2012). Luteolin downregulates TLR4, TLR5, NF-kappaB and p-p38MAPK expression, upregulates the p-ERK expression, and protects rat brains against focal ischemia. Brain Res. 1448, 71–81. 10.1016/j.brainres.2012.02.003 22377454

[B47] Qureshi A. I. Mendelow A. D. Hanley D. F. (2009). Intracerebral haemorrhage. Lancet (London England) 373 (9675), 1632–1644. 10.1016/S0140-6736(09)60371-8 PMC3138486 19427958

[B48] Rochette L. Zeller M. Cottin Y. Vergely C. (2018). Redox functions of heme oxygenase-1 and biliverdin reductase in diabetes. Trends Endocrinol. Metab. 29 (2), 74–85. 10.1016/j.tem.2017.11.005 29249571

[B49] Rojo de la Vega M. Zhang D. D. Wondrak G. T. (2018). Topical bixin confers NRF2-dependent protection against photodamage and hair graying in mouse skin. Front. Pharmacol. 9, 287. 10.3389/fphar.2018.00287 29636694 PMC5880955

[B50] Rusmini P. Cortese K. (2019). Trehalose induces autophagy *via* lysosomal-mediated TFEB activation in models of motoneuron degeneration. Autophagy. 15 (4), 631–651. 10.1080/15548627.2018.1535292 30335591 PMC6526812

[B51] Sawmiller D. Li S. Shahaduzzaman M. Smith A. J. Obregon D. Giunta B. (2014). Luteolin reduces Alzheimer’s disease pathologies induced by traumatic brain injury. Int. J. Mol. Sci. 15 (1), 895–904. 10.3390/ijms15010895 24413756 PMC3907845

[B52] Schiattarella G. G. Hill J. A. (2017). Metabolic control and oxidative stress in pathological cardiac remodelling. Eur. Heart J. 38 (18), 1399–1401. 10.1093/eurheartj/ehw199 27247363

[B53] Selim M. Foster L. D. Moy C. S. Xi G. Hill M. D. Morgenstern L. B. (2019). Deferoxamine mesylate in patients with intracerebral haemorrhage (i-DEF): a multicentre, randomised, placebo-controlled, double-blind phase 2 trial. Lancet Neurol. 18 (5), 428–438. 10.1016/s1474-4422(19)30069-9 30898550 PMC6494117

[B54] Shen H. Chen Z. Wang Y. Gao A. Li H. Cui Y. (2015). Role of neurexin-1beta and neuroligin-1 in cognitive dysfunction after subarachnoid hemorrhage in rats. Stroke 46 (9), 2607–2615. 10.1161/strokeaha.115.009729 26219651 PMC4542569

[B55] Son Y. O. Pratheeshkumar P. Wang Y. Kim D. Zhang Z. Shi X. (2017). Protection from Cr(VI)-induced malignant cell transformation and tumorigenesis of Cr(VI)-transformed cells by luteolin through Nrf2 signaling. Toxicol. Appl. Pharmacol. 331, 24–32. 10.1016/j.taap.2017.04.016 28416455 PMC5568479

[B56] Sosa P. M. de Souza M. A. Mello-Carpes P. B. (2018). Green tea and red tea from camellia sinensis partially prevented the motor deficits and striatal oxidative damage induced by Hemorrhagic stroke in rats. Neural Plast. 2018, 5158724. 10.1155/2018/5158724 30174686 PMC6098885

[B57] Su X. Li T. Liu Z. Huang Q. Liao K. Ren R. (2018). Licochalcone A activates Keap1-Nrf2 signaling to suppress arthritis *via* phosphorylation of p62 at serine 349. Free Radic. Biol. Med. 115, 471–483. 10.1016/j.freeradbiomed.2017.12.004 29233793

[B59] Sun X. Ou Z. Chen R. Niu X. Chen D. Kang R. (2016). Activation of the p62-Keap1-NRF2 pathway protects against ferroptosis in hepatocellular carcinoma cells. Hepatology 63 (1), 173–184. 10.1002/hep.28251 26403645 PMC4688087

[B58] Sun L. Zhang K. Zhai W. Li H. Shen H. Yu Z. (2018). TAR DNA binding Protein-43 loss of function induced by Phosphorylation at S409/410 Blocks autophagic flux and participates in secondary brain injury after intracerebral hemorrhage. Front. Cell Neurosci. 12, 79. 10.3389/fncel.2018.00079 29623031 PMC5874314

[B60] Tan L. Liang C. Wang Y. Jiang Y. Zeng S. Tan R. (2018a). Pharmacodynamic effect of luteolin micelles on alleviating cerebral ischemia reperfusion injury. Pharmaceutics 10 (4), 248. 10.3390/pharmaceutics10040248 PMC6320772 30501051

[B61] Tan X. Liu B. Lu J. Li S. Baiyun R. Lv Y. (2018b). Dietary luteolin protects against HgCl2-induced renal injury *via* activation of Nrf2-mediated signaling in rat. J. Inorg. Biochem. 179, 24–31. 10.1016/j.jinorgbio.2017.11.010 29156292

[B62] Tang X. Wang H. Fan L. Wu X. Xin A. Ren H. (2011). Luteolin inhibits Nrf2 leading to negative regulation of the Nrf2/ARE pathway and sensitization of human lung carcinoma A549 cells to therapeutic drugs. Free Radic. Biol. Med. 50 (11), 1599–1609. 10.1016/j.freeradbiomed.2011.03.008 21402146

[B64] Wang J. Fields J. Zhao C. Langer J. Thimmulappa R. K. Kensler T. W. (2007). Role of Nrf2 in protection against intracerebral hemorrhage injury in mice. Free Radic. Biol. Med. 43 (3), 408–414. 10.1016/j.freeradbiomed.2007.04.020 17602956 PMC2039918

[B63] Wang G. Wang L. Sun X. G. Tang J. (2018a). Haematoma scavenging in intracerebral haemorrhage: from mechanisms to the clinic. J. Cell Mol. Med. 22 (2), 768–777. 10.1111/jcmm.13441 29278306 PMC5783832

[B65] Wang Z. Zhou F. Dou Y. Tian X. Liu C. Li H. (2018b). Melatonin alleviates intracerebral hemorrhage-induced secondary brain injury in rats *via* suppressing apoptosis, inflammation, oxidative stress, DNA damage, and mitochondria injury. Transl. Stroke Res. 9 (1), 74–91. 10.1007/s12975-017-0559-x 28766251 PMC5750335

[B66] Wei C. C. Kong Y. Y. Li G. Q. Guan Y. F. Wang P. Miao C. Y. (2017). Nicotinamide mononucleotide attenuates brain injury after intracerebral hemorrhage by activating Nrf2/HO-1 signaling pathway. Sci. Rep. 7 (1), 717. 10.1038/s41598-017-00851-z 28386082 PMC5429727

[B67] Wu C. Chen J. Yang R. Duan F. Li S. Chen X. (2019). Mitochondrial protective effect of neferine through the modulation of nuclear factor erythroid 2-related factor 2 signalling in ischaemic stroke. Br. J. Pharmacol. 176, 3, 400–415. 10.1111/bph.14537 30414381 PMC6329622

[B68] Xi G. Keep R. F. Hoff J. T. (2006). Mechanisms of brain injury after intracerebral haemorrhage. Lancet Neurol. 5 (1), 53–63. 10.1016/s1474-4422(05)70283-0 16361023

[B69] Xiong J. Wang K. Yuan C. Xing R. Ni J. Hu G. (2017). Luteolin protects mice from severe acute pancreatitis by exerting HO-1-mediated anti-inflammatory and antioxidant effects. Int. J. Mol. Med. 39 (1), 113–125. 10.3892/ijmm.2016.2809 27878246 PMC5179180

[B70] Xu J. Wang H. Ding K. Zhang L. Wang C. Li T. (2014). Luteolin provides neuroprotection in models of traumatic brain injury *via the* Nrf2-ARE pathway. Free Radic. Biol. Med. 71, 186–195. 10.1016/j.freeradbiomed.2014.03.009 24642087

[B71] Xu W. Li F. Liu Z. Xu Z. Sun B. Cao J. (2017). MicroRNA-27b inhibition promotes Nrf2/ARE pathway activation and alleviates intracerebral hemorrhage-induced brain injury. Oncotarget 8 (41), 70669–70684. 10.18632/oncotarget.19974 29050310 PMC5642585

[B72] Yang B. Bai Y. Yin C. Qian H. Xing G. Wang S. (2018a). Activation of autophagic flux and the Nrf2/ARE signaling pathway by hydrogen sulfide protects against acrylonitrile-induced neurotoxicity in primary rat astrocytes. Arch Toxicol. 92, 6, 2093–2108. 10.1007/s00204-018-2208-x 29725710

[B73] Yang S. C. Chen P. J. Chang S. H. Weng Y. T. Chang F. R. Chang K. Y. (2018b). Luteolin attenuates neutrophilic oxidative stress and inflammatory arthritis by inhibiting Raf1 activity. Biochem. Pharmacol. 154, 384–396. 10.1016/j.bcp.2018.06.003 29883707

[B74] Yu Q. Zhang M. Ying Q. Xie X. Yue S. Tong B. (2019). Decrease of AIM2 mediated by luteolin contributes to non-small cell lung cancer treatment. Cell Death Dis. 10 (3), 218. 10.1038/s41419-019-1447-y 30833546 PMC6399355

[B75] Zeng J. Chen Y. (2017). Isoliquiritigenin alleviates early brain injury after experimental intracerebral hemorrhage *via* suppressing ROS- and/or NF-kappaB-mediated NLRP3 inflammasome activation by promoting Nrf2 antioxidant pathway. J. Neuroinflammation. 14, 1, 119. 10.1186/s12974-017-0895-5 28610608 PMC5470182

[B77] Zhang X. Zhang Q. X. Wang X. Zhang L. Qu W. Bao B. (2016). Dietary luteolin activates browning and thermogenesis in mice through an AMPK/PGC1alpha pathway-mediated mechanism. Int. J. Obes. (Lond) 40 (12), 1841–1849. 10.1038/ijo.2016.108 27377953

[B76] Zhang J. X. Xing J. G. Wang L. L. Jiang H. L. Guo S. L. Liu R. (2017). Luteolin Inhibits Fibrillary beta-Amyloid1-40-Induced Inflammation in a Human Blood-Brain Barrier Model by Suppressing the p38 MAPK-Mediated NF-kappaB Signaling Pathways. Molecules 22 (3), 334. 10.3390/molecules22030334 PMC6155314 28245546

[B79] Zhao X. Sun G. Ting S. M. Song S. Zhang J. Edwards N. J. (2015). Cleaning up after ICH: the role of Nrf2 in modulating microglia function and hematoma clearance. J. Neurochem. 133 (1), 144–152. 10.1111/jnc.12974 25328080 PMC4361377

[B78] Zhao L. Chen S. Sherchan P. Ding Y. Zhao W. Guo Z. (2018). Recombinant CTRP9 administration attenuates neuroinflammation *via* activating adiponectin receptor 1 after intracerebral hemorrhage in mice. J. Neuroinflammation. 15, 1, 215. 10.1186/s12974-018-1256-8 30060752 PMC6066941

[B80] Zhou Y. Wang Y. Wang J. Anne Stetler R. Yang Q. W. (2014). Inflammation in intracerebral hemorrhage: from mechanisms to clinical translation. Prog. Neurobiol. 115, 25–44. 10.1016/j.pneurobio.2013.11.003 24291544

