# Supplementary material for: Luteolin Exerts Neuroprotection via Modulation of the p62/Keap1/Nrf2 Pathway in Intracerebral Hemorrhage
Source: Front Pharmacol. 2020 Jan 21;10:1551. doi: 10.3389/fphar.2019.01551 (PMC6985769; doi:10.3389/fphar.2019.01551)

Supplementary Material

**Luteolin** **exerts neuroprotection via modulation of the p62/keap1/Nrf2 pathway in intracerebral hemorrhage**

Xin Tan^#,1^, Yi Yang^#,1^, Jianguo Xu^#,2^, Peng Zhang^2^, Ruming Deng^2^, Yiguang Mao^2^, Jia He^2^, Yibin Chen^2^, Yan Zhang^2^, Jiasheng Ding^2^, Haiying Li^2^, Haitao Shen^2^, Xiang Li*^,2^, Wanli Dong*^,1^, Gang Chen^2^

^1^Department of Neurology, The First Affiliated Hospital of Soochow University, 188 Shizi Street, Suzhou, 215006, Jiangsu Province, China

^2^Department of Neurosurgery & Brain and Nerve Research Laboratory, The First Affiliated Hospital of Soochow University, 188 Shizi Street, Suzhou 215006, China.

**^#^**These authors contributed equally to this work.

*** Correspondence:**

Corresponding Author： Xiang Li, Department of Neurosurgery & Brain and Nerve Research Laboratory, The First Affiliated Hospital of Soochow University, 188 Shizi Street, Suzhou 215006, China. E-mail: xiangli2017@suda.edu.cn; Wanli Dong, Department of Neurology, The First Affiliated Hospital of Soochow University, 188 Shizi Street, Suzhou, 215006, Jiangsu Province, China. E-mail: wanli_dong@126.com

Full images for western blots in Figure 3


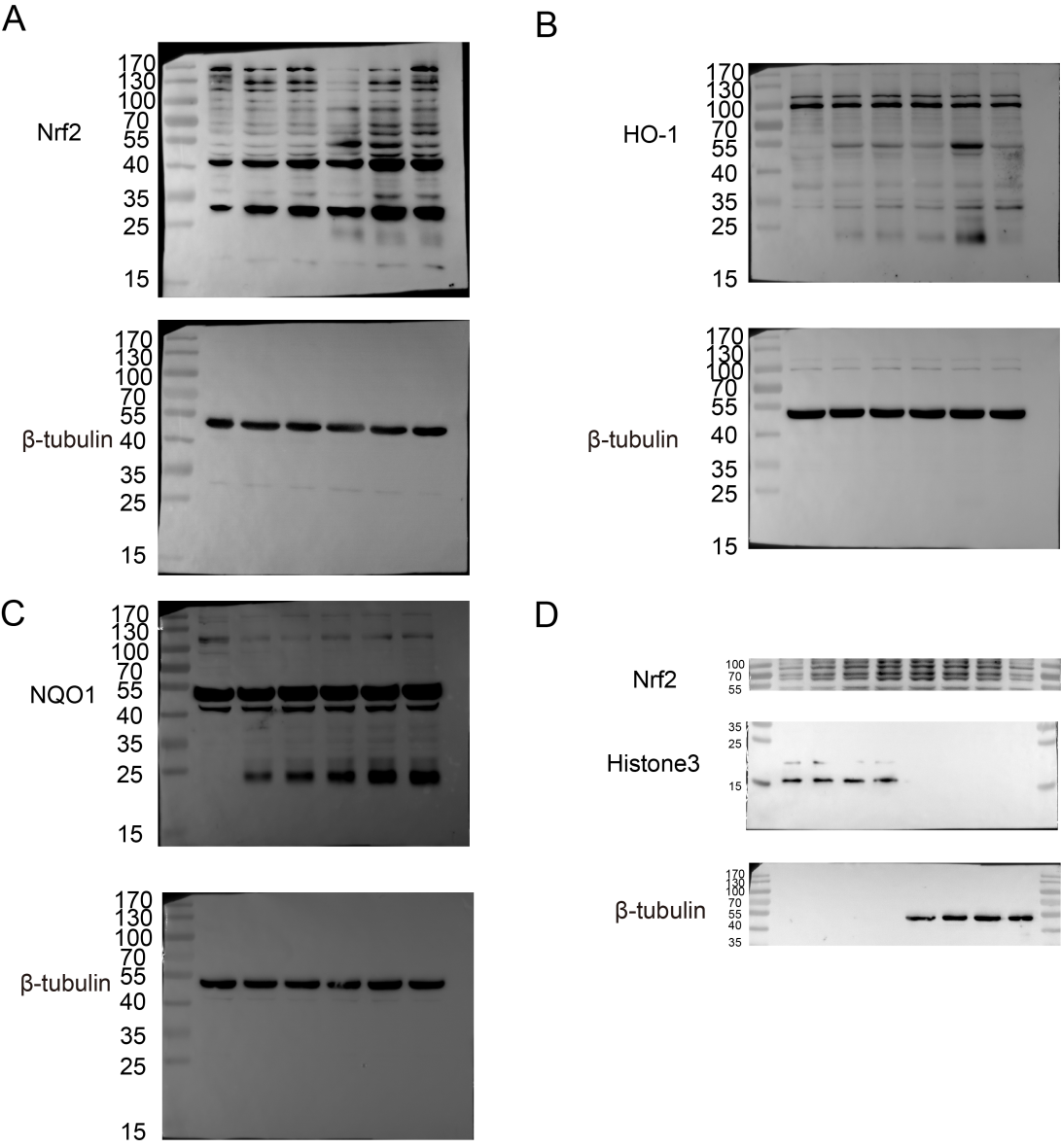


Full images for western blots in Figure 4


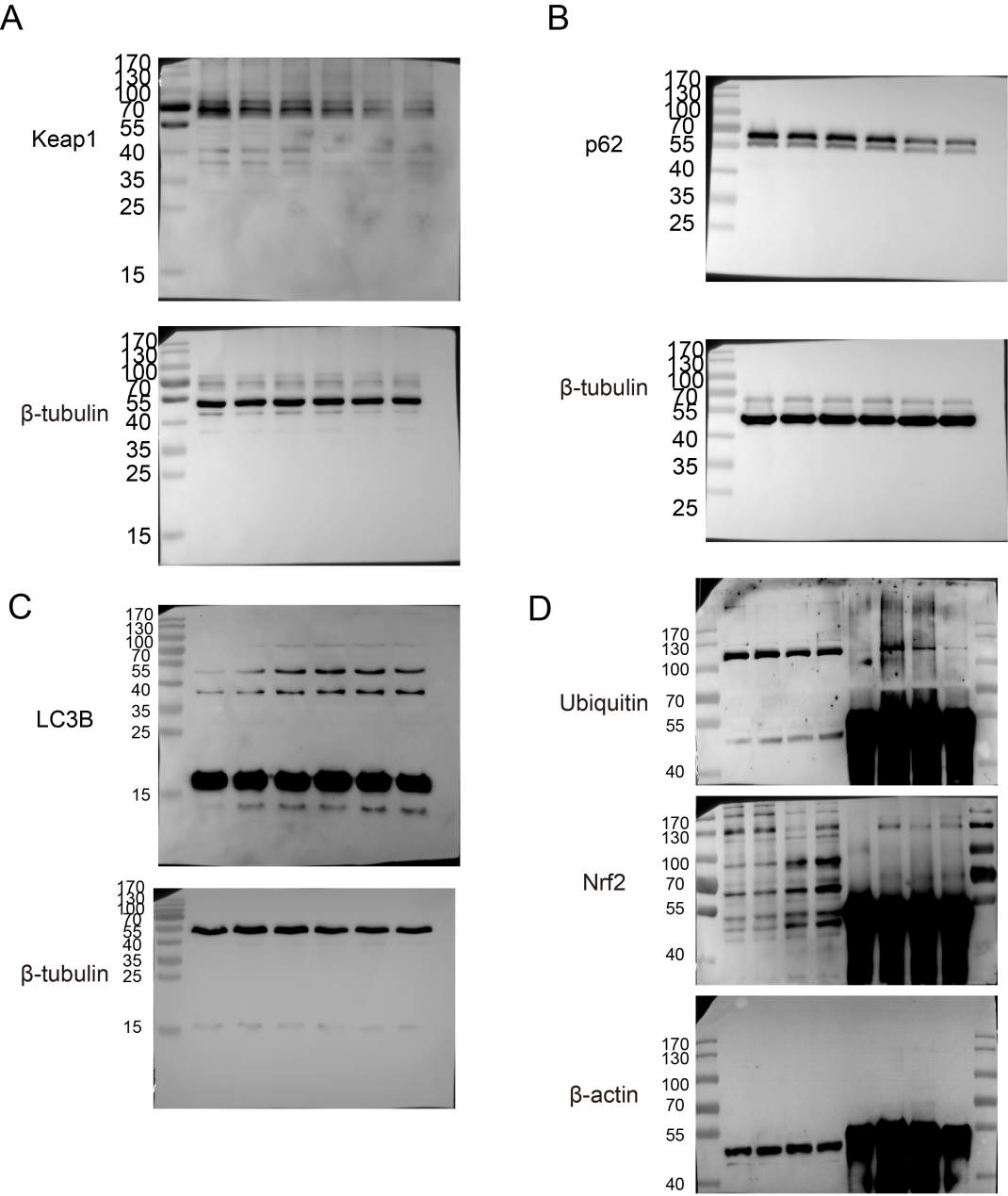


Full images for western blots in Figure 6


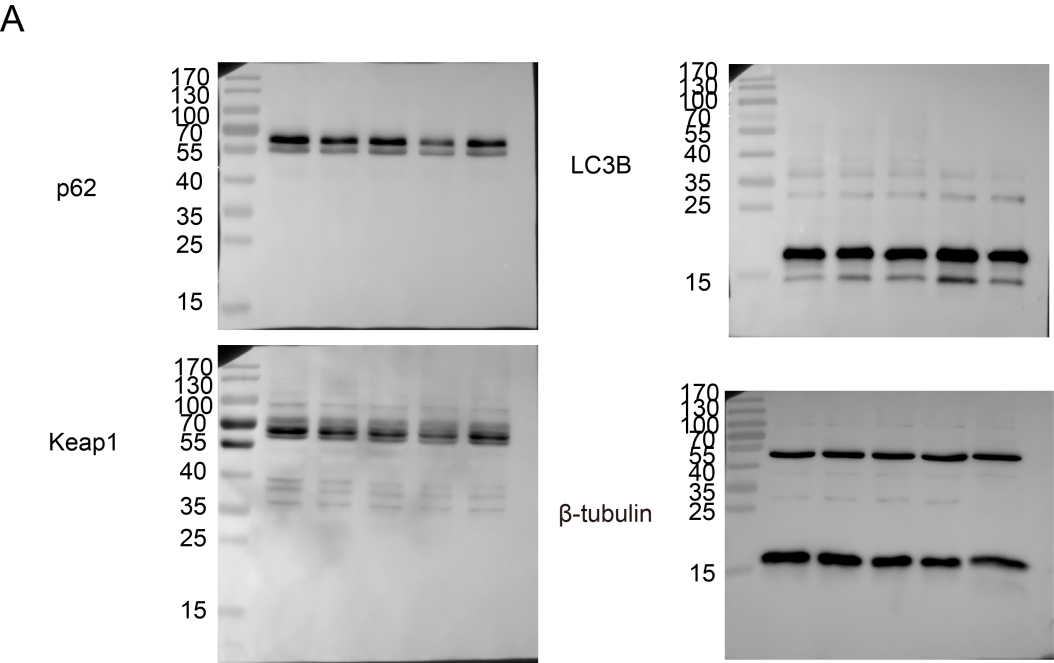

Supplement: Supplementary file 1 [file DataSheet_1.docx]
